# Concomitant Comedications and Survival With First-Line Pembrolizumab in Advanced Non–Small-Cell Lung Cancer

**DOI:** 10.1001/jamanetworkopen.2025.29225

**Published:** 2025-09-10

**Authors:** Adrien Rousseau, Noémie Simon-Tillaux, Stefan Michiels, Lisa Derosa, Ariane Laparra, David Planchard, Jordi Remon, Fabrice Barlesi, Pernelle Lavaud, Maxime Frelaut, Claudia Parisi, Anas Gazzah, Benjamin Besse, Stéphanie Foulon

**Affiliations:** 1 Department of Cancer Medicine, Gustave Roussy, Thoracic Group and International Center for Thoracic Cancers, Paris-Saclay University, Villejuif, France; 2 Oncostat U1018, Institut National de la Santé et de la Recherche Médicale (INSERM), Ligue Contre le Cancer, Paris-Saclay University, Villejuif, France; 3 Biostatistics and Epidemiology Office, Gustave Roussy, Paris-Saclay University, Villejuif, France; 4 Institut National de la Santé et de la Recherche Médicale (INSERM) U1015, Equipe Labellisée, Ligue Nationale Contre le Cancer, Paris-Saclay University, Villejuif, France; 5 Department of Drug Development, Gustave Roussy, Paris-Saclay University, Villejuif, France

## Abstract

**Importance:**

Antibiotics, steroids, and proton pump inhibitors (PPIs) are suspected to decrease the efficacy of immunotherapy.

**Objective:**

To explore the association of comedications with overall survival (OS) in patients with advanced non–small-cell lung cancer (NSCLC).

**Design, Setting, and Participants:**

This nationwide retrospective cohort study used target trial emulations of patients newly diagnosed with NSCLC from January 2015 to December 2022, identified from the French national health care database. Eligible patients were treated with pembrolizumab in a first-line setting and alive 2 months after initiating pembrolizumab. Exclusion criteria included hospitalization for infectious disease, autoimmune disorders, or peptic ulcer disease.

**Exposures:**

Antibiotic and PPI exposures were defined as at least 2 prescriptions 60 days before to 42 days after pembrolizumab start. Steroid exposure was defined as at least 2 prescriptions 30 days before to 30 days after pembrolizumab start.

**Main Outcomes and Measures:**

The primary outcome was OS. Patients exposed were compared with those without exposure, using inverse probability of treatment weighting (IPTW) to adjust for confounding.

**Results:**

Between January 2015, and December 2022, 41 529 patients were treated with first line pembrolizumab for advanced disease (27 826 male [67.0%]; median [range] age, 65 [19-97] years; 14 835 [35.7%] treated with pembrolizumab alone; 26 694 [64.3%] treated with pembrolizumab plus chemotherapy). At treatment initiation, 12 898 (41.9%) patients were exposed to antibiotics, 18 210 (59.1%) to steroids, and 16 783 (53.7%) to PPIs. After IPTW, antibiotics (except for macrolide and penicillin) were associated with shorter OS (hazard ratio [HR], 1.08; 95% CI, 1.05-1.12; *P* < .001), but it varied by antibiotic type. Steroids were not associated with OS (HR, 0.98; 95% CI, 0.95-1.02; *P* = .37); however, there was an interaction with pembrolizumab regimen (ie, pembrolizumab alone or with chemotherapy) (*P *for interaction < .001), and there was a dose-dependent association according to daily prednisone-equivalent dose (*P* for trend < .001). Steroids were associated with worse OS when prescribed at doses greater than 20 mg per day for pembrolizumab alone (*P *for trend = .005) and greater than 30 mg per day for pembrolizumab combined with chemotherapy (*P* for trend < .001). PPIs were associated with worse OS (HR, 1.13; 95% CI, 1.10-1.17; *P* < 001).

**Conclusions and Relevance:**

In this cohort study of patients with advanced NSCLC treated with pembrolizumab, exposure to some classes of antibiotics, to steroids (>20 mg per day of prednisone equivalent), and to PPIs was associated with worse OS, indicating that comedications should be monitored carefully in patients with immunotherapy.

## Key Points

**Question:**

Are comedications at pembrolizumab start associated with worse overall survival (OS) in patients with advanced non–small-cell lung cancer (NSCLC)?

**Findings:**

In this cohort study of 41 529 patients with NSCLC, after adjustment for several confounders, exposure to antibiotics (with the exception of macrolide and penicillin), to high doses of steroids (>20 mg per day of equivalent prednisone), and to proton pump inhibitors at the beginning of pembrolizumab treatment were associated with worse OS.

**Meaning:**

These findings suggest that comedications should be monitored carefully in patients with immunotherapy.

## Teaser

This cohort study using a target trial emulation approach examines associations of comedications (antibiotics, steroids, and proton pump inhibitors) with survival among patients in France with non–small cell lung cancer (NSCLC) who received pembrolizumab as a first-line treatment.

## Introduction

Immune checkpoint blockers have revolutionized the prognosis of patients with advanced non–small cell lung cancer (NSCLC). Pembrolizumab was approved by the European Medicines Agency for untreated patients with advanced NSCLC and high programmed death-ligand 1 (PD-L1) expression (≥50%) in January 2017^1^ and in combination with chemotherapy regardless of PD-L1 expression since February 2019.^2^ The impact of comedications on the outcome of patients undergoing immunotherapy is a dynamic area of research, particularly regarding corticosteroids^3^ because of their immunosuppressive properties, antibiotics,^4^ and proton pump inhibitors (PPIs)^5^ because of their potential impact on gut microbiota.^6^ Although the exposure period to antibiotics varies across studies, 2 meta-analyses^7,8^ on more than 40 000 patients found that antibiotics prescriptions were associated with poorer outcomes in patients treated by immunotherapy or chemo-immunotherapy. Regarding PPIs, the exposure period is also not consistently defined and sometimes not even specified. A meta-analysis^9^ demonstrated that concomitant PPI administration was associated with lower progression-free survival (PFS) and overall survival (OS). A retrospective study^5^ on patients with lung cancer observed that those exposed to PPIs at the first dose of immunotherapy had lower survival, but this was not the case for patients treated with chemotherapy combined with immunotherapy. However, a pooled analysis^10^ of nearly 13 000 patients with advanced lung cancer found that PPI use was associated with shorter PFS and OS in patients undergoing chemotherapy, targeted therapy, and immunotherapy. Conversely, a post hoc analysis of a phase II trial^11^ did not find an association of PPI use on the day of nivolumab initiation with OS or PFS. Exposure to corticosteroids is also inconsistently defined in the literature, but the at-risk period seems to be just before and immediately after the initiation of immunotherapy.^12^ A retrospective study^13^ showed that corticosteroid exposure at the start of immunotherapy was associated with lower PFS and OS but another study^3^ found that patients exposed to steroids had a longer PFS and OS. Most of these studies presented small sample sizes, imperfect reporting and confounding, and immortal time biases. Recently, target trial emulation has been proposed as a methodological approach that can improve confidence in observational analyses by making the causal question more transparent and helping to mitigate biases.^14^ Using empirical data from a large claim database,^15^ this study aimed to explore the association of antibiotic, PPI, or steroid exposure at pembrolizumab start with OS in patients with untreated advanced NSCLC using a target trial emulation approach.

## Methods

### Study Design

This cohort study uses a target trial emulation approach. eTable 1 in Supplement 1 outlines the key components of the ideal target trials protocols, that is, the randomized experiments that would have been conducted to answer the research questions (one by comedication), and their emulations in the observational setting, as recommended by the Transparent Reporting of Observational Studies Emulating a Target Trial (TARGET) guidelines.^16^

### Data Source

The French National Health Data System (SNDS) includes demographic and medical data on most outpatient services reimbursed by the National Health Insurance since 2006. The ATHENA cohort included all patients with an incident diagnosis of lung cancer in France from January 1, 2015, to December 31, 2022, and was previously described elsewhere.^17^ Detailed information is available in the eMethods in Supplement 1.

### Regulatory Approval and Ethical Aspects

Gustave Roussy is certified to have permanent access to SNDS, so this study only required a declaration prior to data extraction on the Health Data Hub online platform. The SNDS is a strictly anonymous database, so informed consent was not needed.

### Eligibility Criteria

In the emulated trials, inclusion criteria were any hospitalization or any expenses for a long-term condition identified by *International Statistical Classification of Diseases and Related Health Problems, Tenth Revision (ICD-10)* lung cancer codes, being older than 18 years, and being treated with pembrolizumab in first-line setting. The exclusion criteria were at least 1 onset of a hospitalization with a lung cancer code during the 5 previous years preceding the study period and same-sex twins or changes in insurance plans (potential source of identification’s error). Patients with another cancer diagnosis, identified the previous year prior to the date index, were also excluded to avoid misclassification bias (lung metastasis of another cancer coded as lung cancer). To respect the positivity assumption,^18^ we added specific exclusion criteria for the emulated trial: being hospitalized for infectious disease 2 months prior to the immunotherapy start for antibiotics’ analysis (emulated trial 1), being hospitalized for autoimmune disease or organ transplantation 12 months prior to the immunotherapy start for steroids analysis (emulated trial 2), and being hospitalized for esophagitis, gastritis, duodenitis, or ulcer, or having a fibroscopy 6 months prior to the immunotherapy start for PPI analysis (emulated trial 3). To limit immortal time bias,^19^ an inclusion criterion was to be alive 2 months after the first pembrolizumab, and follow-up was reset at this time point in the analysis (landmark time or baseline). Important variables are defined in eTable 2 in Supplement 1.

### Exposures

Antibiotic exposure was defined as at least 2 prescriptions with Anatomical Therapeutic Chemical (ATC) code J01 60 days before to 42 days after pembrolizumab start. PPI exposure was defined as at least 2 prescriptions with ATC code A02BC, 60 days before to 42 days after pembrolizumab start. These time frames were chosen by microbiota experts as the most at risk for affecting immune response.^6,20^ Steroid exposure was defined as at least 2 prescriptions with ATC code H02, 30 days before to 30 days after pembrolizumab start. Steroid dose was defined as the mean daily prescribed dose during this period, converted in milligrams of oral prednisone. For all the analyses, the comparator was patients without the exposure of interest.

### Outcome

For each of the 3 exposures, the primary outcome was OS. OS was defined as the time between landmark time and death from any cause.

### Statistical Analysis

Time-to-event end points were calculated using the propensity-weighted Kaplan-Meier estimators. Patients without events were censored at the last care reimbursement date. The cut-off date for the analysis was June 30, 2023. Missing data, affecting less than 10% of sample, and concerning almost exclusively the deprivation index, were excluded. To prevent the introduction of colliders and to assure the required adjustment on confounders, we modeled our hypotheses with causal directed acyclic graphs using Dagitty software version 3.1 (eFigure 1 in Supplement 1). To account for potential confounding, we used a Cox model weighted by the inverse probability of treatment (propensity score). The detailed model is explained in the eMethods in Supplement 1. The proportional hazards assumption was verified with Schoenfeld residuals. Positivity assumptions were verified by plotting propensity score by exposure groups (exposed and unexposed) (eFigure 2 in Supplement 1), and the conditional exchangeability assumption was verified evaluating absolute standardized mean differences depicted in Love plots (eFigure 3 in Supplement 1). Because variance can be biased with weighting,^21^ all 95% CIs were estimated by a bootstrap procedure (1000 samples). In addition to hazard ratios (HRs), we also calculated the absolute difference in probabilities of survival at 24 months, a valid estimand regardless of proportional hazard. To estimate these probabilities, we used a Kaplan-Meier estimator weighted by propensity score, and 95% CIs were also estimated by a bootstrap procedure. The timing of 24 months was considered relevant because it matches the complete duration of treatment for immunotherapy in NSCLC.^1^ In addition, we looked at heterogeneity in the association with exposure according to the regimen of immunotherapy (alone or in combination with chemotherapy) and an interaction term was introduced in the models. If an interaction was found, all the subsequent analyses (type or dose of exposure) were performed in 2 separated populations (immunotherapy alone and chemo-immunotherapy). Other sensitivity analyses are explained in the eMethods in Supplement 1. All statistical tests were 2-tailed, with a type I error of 5%. Statistical analyses were performed with SAS version 9.4 (SAS Institute) and R software version 4.1.2 (R Project for Statistical Computing).

## Results

### Population

Between January 1, 2015, and December 31, 2022, 41 529 patients were treated with first line pembrolizumab for an advanced disease (eFigure 4 and eTable 3 in Supplement 1). Of these patients, 27 826 (67.0%) were male, median (range) age was 65 (19-97) years, 14 835 (35.7%) were treated with pembrolizumab alone, and 26 694 (64.3%) were treated with pembrolizumab plus chemotherapy (Table). Median (IQR) survival was 24.8 (24.5-25.1) months. At treatment initiation, 12 898 patients (41.9%) were exposed to antibiotics, 18 210 (59.1%) to steroids, and 16 783 (53.7%) to PPIs (Table). With regards to absolute standardized mean differences, patients exposed to antibiotics were more frequently exposed to steroids and PPIs, had more chronic respiratory disease, and received more painkillers and anti-inflammatory prescriptions. Patients exposed to steroids were younger but were more frequently exposed to antibiotics and PPIs; were more frequently treated with chemo-immunotherapy; and had more antiepileptic, painkiller, and radiation therapy prescriptions. Patients exposed to PPIs were more often exposed to steroids and antibiotics; were more frequently treated with chemo-immunotherapy; and had more antiepileptic, painkiller, anti-inflammatory, antiplatelet, β-blocker, and radiation therapy prescriptions. After weighting, the characteristics were well balanced (eFigure 3 in Supplement 1).

**Table.** Patient Characteristics

Characteristic	Participants, No. (%) (N = 41 529)					
	Antibiotics exposure (n = 12 898)	No exposure to antibiotics (n = 17 846)	Exposure to steroids (n = 18 210)	No exposure to steroids (n = 12 609)	Exposure to PPI (n = 16 783)	No exposure to PPI (n = 14 494)
Sex						
Female	4469 (34.6)	5856 (32.8)	6462 (35.5)	3830 (30.4)	5680 (33.8)	4779 (33.0)
Male	8429 (65.4)	11 990 (67.2)	11 748 (64.5)	8779 (69.6)	11 103 (66.2)	9715 (67.0)
Age at diagnosis, y						
Mean (SD)	64.2 (9.75)	65.1 (9.78)	63.2 (9.29)	66.8 (10.10)	64.5 (9.73)	64.9 (9.85)
Median (range)	65.0 (25.0-95.0)	65.0 (24.0-97.0)	64.0 (24.0-92.0)	67.0 (26.0-97.0)	65.0 (24.0-93.0)	65.0 (24.0-97.0)
Age category, y						
18-49.99	899 (7.0)	1102 (6.2)	1424 (7.8)	600 (4.8)	1149 (6.8)	903 (6.2)
50-59.99	3058 (23.7)	3824 (21.4)	4550 (25.0)	2362 (18.7)	3824 (22.8)	3190 (22.0)
60-59.99	5120 (39.7)	7005 (39.3)	7542 (41.4)	4609 (36.6)	6617 (39.4)	5693 (39.3)
70-79.99	3119 (24.2)	4660 (26.1)	4104 (22.5)	3663 (29.1)	4222 (25.2)	3694 (25.5)
80-97.99	702 (5.4)	1255 (7.0)	590 (3.2)	1375 (10.9)	971 (5.8)	1014 (7.0)
Year of diagnosis						
2015-2016	306 (2.4)	461 (2.6)	365 (2.0)	400 (3.2)	441 (2.6)	345 (2.4)
2017-2018	1772 (13.7)	2541 (14.2)	1654 (9.1)	2699 (21.4)	2275 (13.6)	2152 (14.8)
2019-2020	4393 (34.1)	6370 (35.7)	6251 (34.3)	4542 (36.0)	5853 (34.9)	5071 (35.0)
2021-2022	6427 (49.8)	8474 (47.5)	9940 (54.6)	4968 (39.4)	8214 (48.0)	6926 (47.8)
Type of hospital						
High volume	5512 (42.7)	7457 (41.8)	7970 (43.8)	5025 (39.9)	6938 (41.3)	6260 (43.2)
Intermediate volume	5564 (43.1)	7670 (43.0)	7843 (43.1)	5406 (42.9)	7308 (43.5)	6146 (42.4)
Low volume	1822 (14.1)	2719 (15.2)	2397 (13.2)	2178 (17.3)	2537 (15.1)	2088 (14.4)
Deprivation index						
First quintile	2203 (17.1)	2954 (16.6)	3071 (16.9)	2096 (16.6)	2819 (16.8)	2418 (16.7)
Second quintile	2614 (20.3)	3673 (20.6)	3736 (20.5)	2547 (20.2)	3416 (20.4)	2960 (20.4)
Third quintile	2638 (20.5)	3715 (20.8)	3761 (20.7)	2605 (20.7)	3417 (20.4)	3044 (21.0)
Fourth quintile	2743 (21.3)	3883 (21.8)	3909 (21.5)	2736 (21.7%)	3611 (21.5)	3142 (21.7)
Fifth quintile	2700 (20.9)	3621 (20.3)	3733 (20.5)	2625 (20.8)	3520 (21.0)	2930 (20.2)
Strategy						
Pembrolizumab alone	4141 (32.1)	6306 (35.3)	2887 (15.9)	7648 (60.7)	5202 (31.0)	5510 (38.0)
Pembrolizumab plus chemotherapy	8757 (67.9)	11 540 (64.7)	15 323 (84.1)	4961 (39.3)	11 581 (69.0)	8984 (62.0)
Pemetrexed treatment						
Without pemetrexed	6313 (48.9)	9147 (51.3)	5804 (31.9)	9759 (77.4)	7888 (47.0)	7912 (54.6)
With pemetrexed	6585 (51.1)	8699 (48.7)	12 406 (68.1)	2850 (22.6)	8895 (53.0)	6582 (45.4)
Type of first hospitalization						
Day hospital	9811 (76.1)	13 702 (76.8)	13 856 (76.1)	9580 (76.0)	12502 (74.5)	11 267 (77.7)
Inpatient hospitalization	3087 (23.9)	4144 (23.2)	4354 (23.9)	3029 (24.0)	4281 (25.5)	3227 (22.3)
Radiation therapy at baseline						
No	12 056 (93.5)	16 391 (91.8)	16 571 (91.0)	11 971 (94.9)	15 197 (90.6)	13 747 (94.8)
Yes	842 (6.5)	1455 (8.2)	1639 (9.0)	638 (5.1)	1586 (9.5)	747 (5.2)
Antiepileptic at baseline						
No	12 067 (93.6)	16 658 (93.3)	16 625 (91.3)	12 168 (96.5)	15 317 (91.3)	13 902 (95.9)
Yes	831 (6.4)	1188 (6.7)	1585 (8.7)	441 (3.5)	1466 (8.7)	592 (4.1)
Exposure to antibiotics at baseline						
No	0	17 846 (100)	9623 (52.8)	8045 (63.8)	8832 (52.6)	9081 (62.7)
Yes	12 898 (100)	0	8587 (47.2)	4564 (36.2)	7951 (47.4)	5413 (37.3)
Exposure to steroid at baseline						
No	4421 (34.3)	8067 (45.2)	0	12 609 (100)	5418 (32.3)	7355 (50.7)
Yes	8477 (65.7)	9779 (54.8)	18 210 (100)	0	11 365 (67.7)	7139 (49.3)
Exposure to PPI at baseline						
No	5210 (40.4)	9024 (50.6)	7056 (38.7)	7249 (57.5)	0	14 494 (100)
Yes	7688 (59.6)	8822 (49.4)	11 154 (61.3)	5360 (42.5)	16 783 (100)	0
Severe myocardial infarction at diagnosis						
No	12 811 (99.3)	17 704 (99.2)	18 093 (99.4)	12 501 (99.1)	16 618 (99.0)	14 431 (99.6)
Yes	87 (0.7)	142 (0.8)	117 (0.6)	108 (0.9)	165 (1.0)	63 (0.4)
Severe heart failure at diagnosis						
No	12 826 (99.4)	17 713 (99.3)	18 138 (99.6)	12 470 (98.9)	16 651 (99.2)	14 412 (99.4)
Yes	72 (0.6)	133 (0.7)	72 (0.4)	139 (1.1)	132 (0.8)	82 (0.6)
Severe cerebrovascular disease at diagnosis						
No	12 772 (99.0)	17 620 (98.7)	18 039 (99.1)	12 427 (98.6)	16 592 (98.9)	14 326 (98.8)
Yes	126 (1.0)	226 (1.3)	171 (0.9)	182 (1.4)	191 (1.1)	168 (1.2)
Severe peripheral artery disease at diagnosis						
No	12 752 (98.9)	17 617 (98.7)	18 010 (98.9)	12 433 (98.6)	16 543 (98.6)	14 347 (99.0)
Yes	146 (1.1)	229 (1.3)	200 (1.1)	176 (1.4)	240 (1.4)	147 (1.0)
Chronic respiratory disease at diagnosis						
No	8690 (67.4)	13 328 (74.7)	13 225 (72.6)	8800 (69.8)	11 775 (70.2)	10 592 (73.1)
Yes	4208 (32.6)	4518 (25.3)	4985 (27.4)	3809 (30.2)	5008 (29.8)	3902 (26.9)
Diabetes at diagnosis						
No	11 292 (87.5)	15 559 (87.2)	16 193 (88.9)	10 711 (84.9)	14 402 (85.8)	12 895 (89.0)
Yes	1606 (12.5)	2287 (12.8)	2017 (11.1)	1898 (15.1)	2381 (14.2)	1599 (11.0)
Severe liver disease at diagnosis						
No	12 892 (100.0)	17 842 (100.0)	18 203 (100.0)	12 606 (100.0)	16 775 (100.0)	14 492 (100.0)
Yes	6 (<0.0)	4 (<0.0)	7 (<0.0)	3 (<0.0)	8 (<0.0)	2 (<0.0)
Severe kidney disease at diagnosis						
No	12 872 (99.8)	17 801 (99.7)	18 189 (99.9)	12 563 (99.6)	16 738 (99.7)	14 468 (99.8)
Yes	26 (0.2)	45 (0.3)	21 (0.1)	46 (0.4)	45 (0.3)	26 (0.2)
Renin-angiotensin system inhibitor at diagnosis						
No	8424 (65.3)	11 463 (64.2)	12 202 (67.0)	7736 (61.4)	10 360 (61.7)	9863 (68.0)
Yes	4474 (34.7)	6383 (35.8)	6008 (33.0)	4873 (38.6)	6423 (38.3)	4631 (32.0)
Antiplatelet at diagnosis						
No	9060 (70.2)	12 356 (69.2)	13 126 (72.1)	8319 (66.0)	11 009 (65.6)	10 750 (74.2)
Yes	3838 (29.8)	5490 (30.8)	5084 (27.9)	4290 (34.0)	5774 (34.4)	3744 (25.8)
Lipid lowering drug at diagnosis						
No	8240 (63.9)	11 283 (63.2)	11 966 (65.7)	7592 (60.2)	9982 (59.5)	9870 (68.1)
Yes	4658 (36.1)	6563 (36.8)	6244 (34.3)	5017 (39.8)	6801 (40.5)	4624 (31.9)
Anticoagulant at diagnosis						
No	11 043 (85.6)	15 395 (86.3)	15 772 (86.6)	10 737 (85.2)	14 152 (84.3)	12 723 (87.8)
Yes	1855 (14.4%	2451 (13.7)	2438 (13.4)	1872 (14.8)	2631 (15.7)	1771 (12.2)
Diurectic at diagnosis						
No	11 513 (89.3)	15 932 (89.3)	16 480 (90.5)	11 014 (87.4)	14 701 (87.6)	13 190 (91.0)
Yes	1385 (10.7)	1914 (10.7)	1730 (9.5)	1595 (12.6)	2082 (12.4)	1304 (9.0)
β-blocker at diagnosis						
No	10 223 (79.3)	13 948 (78.2)	14 631 (80.3)	9599 (76.1)	12 725 (75.8)	11 851 (81.8)
Yes	2675 (20.7)	3898 (21.8)	3579 (19.7)	3010 (23.9)	4058 (24.2)	2643 (18.2)
Nonsteroidal anti-inflammatory at diagnosis						
No	8062 (62.5)	12 247 (68.6)	11 051 (60.7)	9414 (74.7)	9982 (59.5)	10 691 (73.8)
Yes	4836 (37.5)	5599 (31.4)	7159 (39.3)	3195 (25.3)	6801 (40.5)	3803 (26.2)
Antipsychotic at diagnosis						
No	12 507 (97.0)	17 198 (96.4)	17 694 (97.2)	12 079 (95.8)	16 249 (96.8)	13 973 (96.4)
Yes	391 (3.0)	648 (3.6)	516 (2.8)	530 (4.2)	534 (3.2)	521 (3.6)
Antidepressant at diagnosis						
No	10 735 (83.2)	14 937 (83.7)	15 232 (83.6)	10 550 (83.7)	13 651 (81.3)	12 470 (86.0)
Yes	2163 (16.8)	2909 (16.3)	2978 (16.4)	2059 (16.3)	3132 (18.7)	2024 (14.0)
Thyroid hormone replacement drug at diagnosis						
No	11 972 (92.8)	16 570 (92.9)	16 983 (93.3)	11 634 (92.3)	15 506 (92.4)	13 542 (93.4)
Yes	926 (7.2)	1276 (7.2)	1227 (6.7)	975 (7.7)	1277 (7.6)	952 (6.6)
Painkiller at diagnosis						
No	2413 (18.7)	4746 (26.6)	3677 (20.2)	3579 (28.4)	2803 (16.7)	4469 (30.8)
Yes	10485 (81.3)	13100 (73.4)	14533 (79.8)	9030 (71.6)	13980 (83.3)	10025 (69.2)
Opiate substitute drug at diagnosis						
No	12786 (99.1)	17708 (99.2)	18054 (99.1)	12509 (99.2)	16639 (99.1)	14378 (99.2)
Yes	112 (0.9)	138 (0.8)	156 (0.9)	100 (0.8)	144 (0.9)	116 (0.8)

Abbreviation: PPI, proton pump inhibitor.

### Antibiotics Analysis

**Figure 1.**
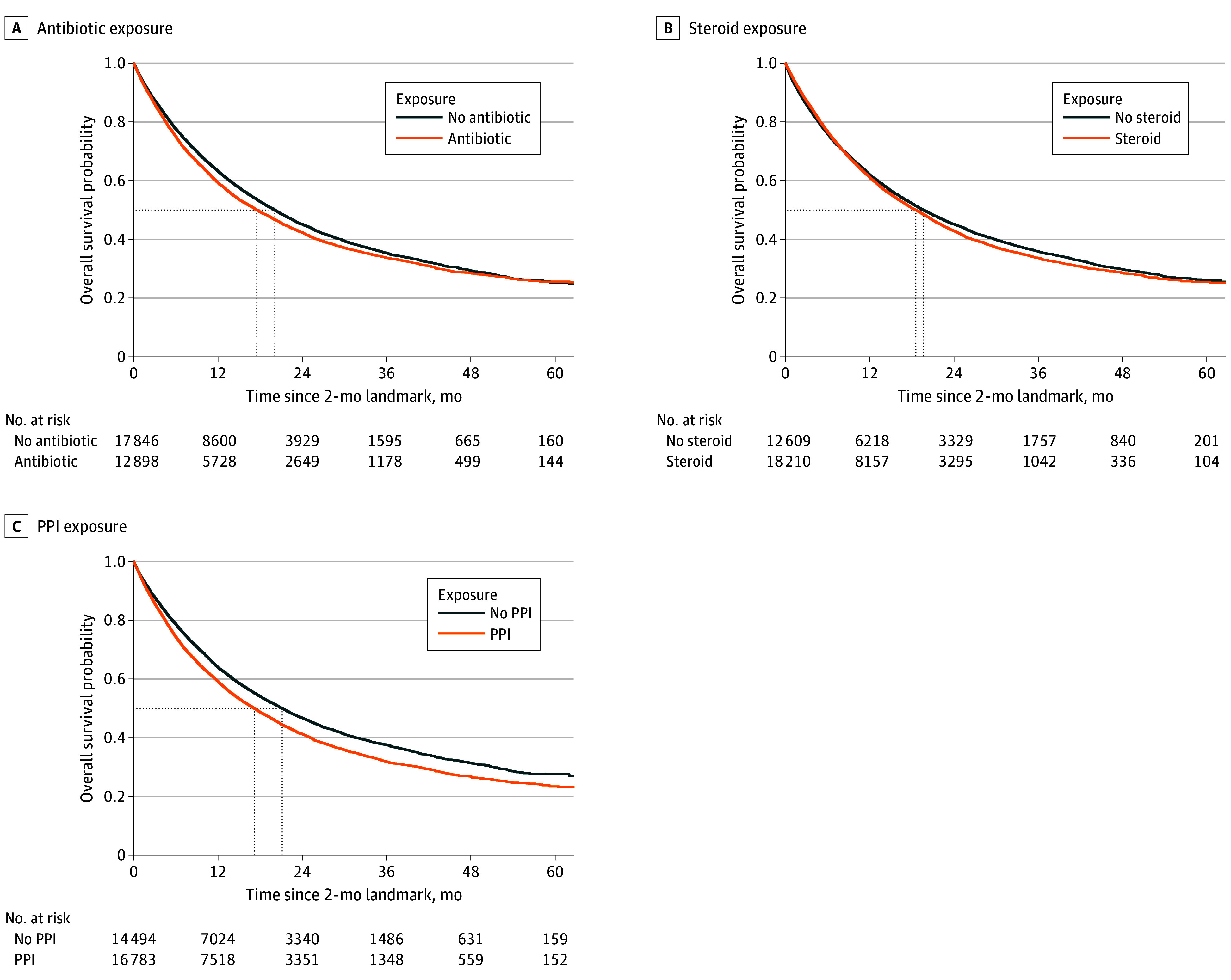
Weighted Distribution by Antibiotc, Steroid, or Proton Pump Inhibitor (PPI) Exposure Numbers at risk are also weighted and may differ from eFigure 4 in Supplement 1.

**Figure 2.**
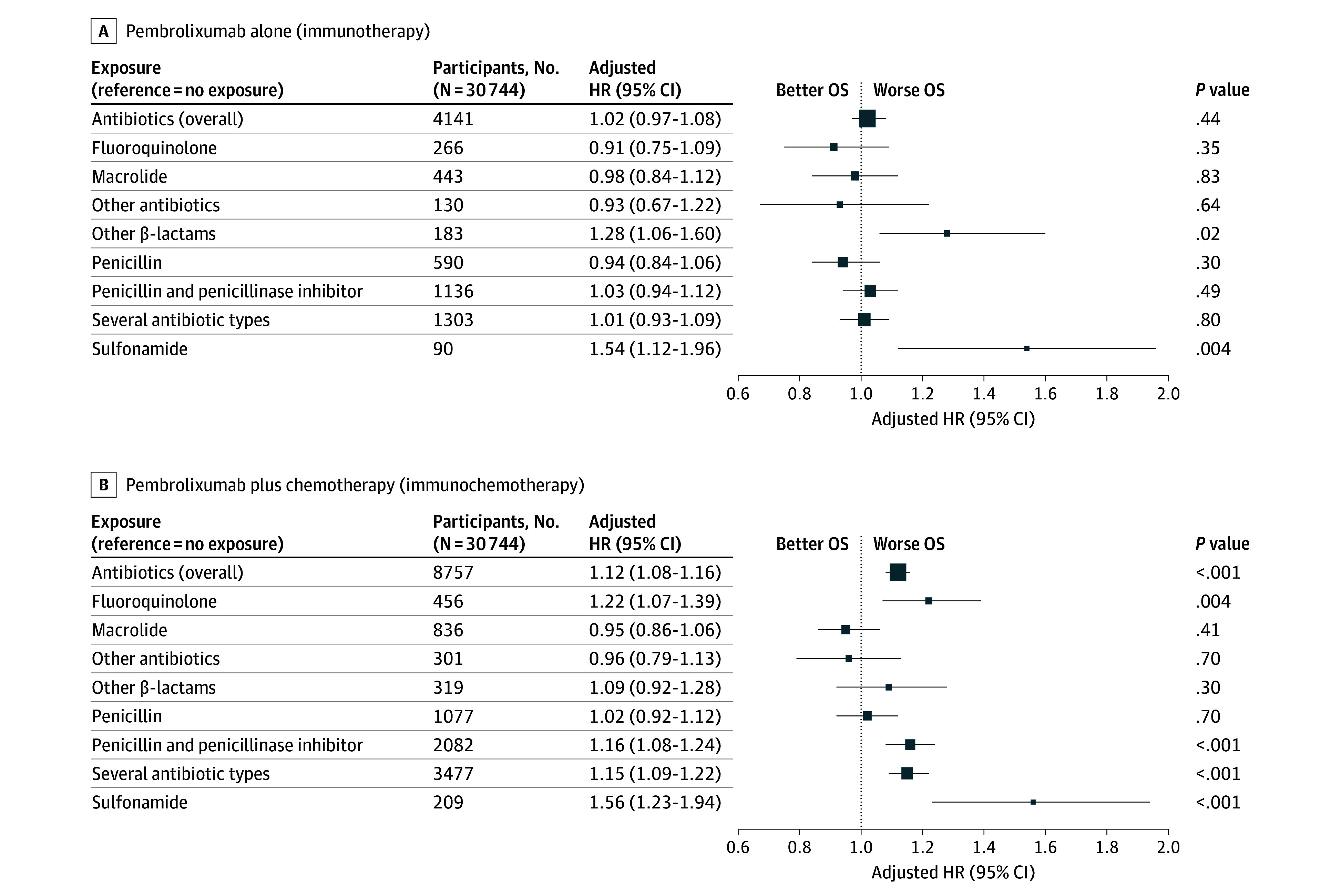
Assocications of Overall Survival (OS) With Antibiotic Exposure Among Those Receiving Pembrolizumab Alone and Pembrolizumab Plus Chemotherapy HR indicates hazard ratio.

Antibiotic exposure was associated with worse OS (HR, 1.08; 95% CI, 1.05 to 1.12; *P* < .001) (Figure 1 and Figure 2) and with decreased survival at 24 months (absolute difference in probabilities of survival at 24 months, −2.53%; 95% CI, −3.82% to −1.22%) (eTable 4 in Supplement 1). However, there was a significant interaction with the regimen of immunotherapy alone or combined with chemotherapy (*P *for interaction = .007), thus we differentiated the analyses in 2 separate populations. In the pembrolizumab alone population, antibiotic exposure was not significantly associated with OS (HR, 1.02; 95% CI, 0.97 to 1.08; *P* = .44; absolute difference in probabilities of survival at 24 months, −0.77%; 95% CI, −2.89% to 1.23%). However, results varied by type of antibiotics, and some were associated with worse OS (other β lactams: HR, 1.28; 95% CI, 1.06 to 1.60; *P* = .02; absolute difference in probabilities of survival at 24 months, −8.47%; 95% CI, −18.01% to −1.49%; sulfonamides: HR, 1.54; 95% CI, 1.12 to 1.96; *P* = .004; absolute difference in probabilities of survival at 24 months, −19.30%; 95% CI, −31.12% to −7.09%). Fluoroquinolones, macrolides, other antibiotics, penicillins, penicillins plus penicillinase inhibitors, and the combination of several antibiotic types were not significantly associated with survival. In the pembrolizumab plus chemotherapy population, antibiotic exposure was associated with worse OS (HR, 1.12; 95% CI, 1.08 to 1.16; *P* < .001; absolute difference in probabilities of survival at 24 months, −3.65%; 95% CI, −5.43% to −2.04%). The types of antibiotics associated with worse OS were fluoroquinolones (HR, 1.22; 95% CI, 1.07 to 1.39; *P* = .004; absolute difference in probabilities of survival at 24 months, −8.55%; 95% CI, −15.21% to −2.94%), penicillins plus penicillinase inhibitors (HR, 1.16; 95% CI, 1.08-1.24; *P* < .001; absolute difference in probabilities of survival at 24 months, −4.69%; 95% CI, −7.71% to −1.84%), combination of several antibiotic types (HR, 1.15; 95% CI, 1.09 to 1.22; *P* < .001; absolute difference in probabilities of survival at 24 months, −4.61%; 95% CI, −7.18% to −2.34%), and sulfonamides (HR, 1.56; 95% CI, 1.23 to 1.94; *P* < .001; absolute difference in probabilities of survival at 24 months, −12.73%; 95% CI, NA to −5.44). Macrolides, other antibiotics, penicillins and other β lactams were not associated with survival.

### Steroids Analysis

**Figure 3.**
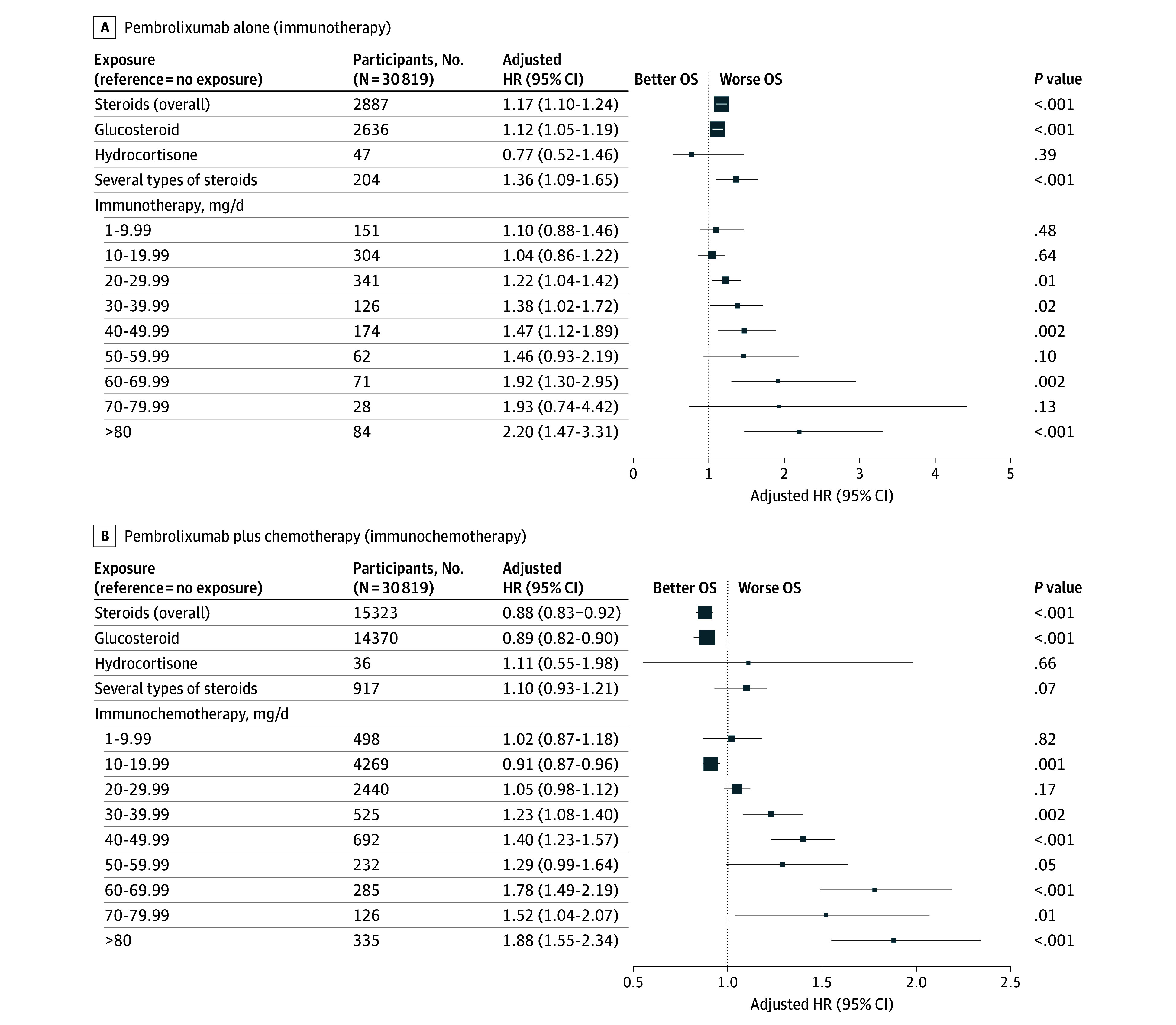
Associations of Overall Survival (OS) With Steroid Exposure Among Those Receiving Pembrolizumab Alone and Pembrolizumab Plus Chemotherapy HR indicates hazard ratio.

Steroid exposure was not significantly associated with survival (HR, 0.98; 95% CI, 0.95 to 1.02; *P* = .37; absolute difference in probabilities of survival at 24 months, 0.00%; 95% CI, −1.54% to 1.72%) (Figure 1, Figure 3, and eTable 4 in Supplement 1). However, there was an interaction with the regimen of immunotherapy (P for interaction = .001), thus we differentiated the analyses in 2 separate populations. In the pembrolizumab alone population, steroid exposure was associated with worse OS (HR, 1.17; 95% CI, 1.10 to 1.24; *P* < .001; absolute difference in probabilities of survival at 24 months, −6.00%; 95% CI, −8.42% to −3.44%). Exposure to glucosteroids (HR, 1.12; 95% CI, 1.05 to 1.19; *P* < .001; absolute difference in probabilities of survival at 24 months, −4.44%; 95% CI, −7.36% to −2.03%) and to several steroid types (HR, 1.36; 95% CI, 1.09-1.65; *P* < .001; absolute difference in probabilities of survival at 24 months, −15.30%; 95% CI, −18.03 to −2.06) was associated with worse OS. The association with OS was dependent on the steroid dose. A negative association with OS was observed when prescribed more than 20 mg per day, with a dose response association (*P *for trend = .005) (Figure 3 and eFigure 5 in Supplement 1). In the pembrolizumab plus chemotherapy population, steroid exposure was associated with better OS (HR, 0.88; 95% CI, 0.83 to 0.92; *P* < .001; absolute difference in probabilities of survival at 24 months, 3.53%; 95% CI, 1.39% to 5.77%). Exposure to glucosteroids (whatever the dose) was associated with better OS (HR, 0.89; 95% CI, 0.82 to 0.90; *P* < .001; absolute difference in probabilities of survival at 24 months, 2.39%; 95% CI, 1.38% to 6.63%). Regarding prednisone-equivalent dose, the only subgroup of patients with a beneficial association with OS was those who took 10 to 20 mg per day (HR, 0.91; 95% CI, 0.87 to 0.96; *P* < .001; absolute difference in probabilities of survival at 24 months, 1.54%; 95% CI, −0.93% to 3.74%). There was also a dose-response association and a negative dose association for prednisone equivalents greater than 30 mg per day (*P *for trend < .001) (Figure 3 and eFigure 5 in Supplement 1).

### PPI Analysis

**Figure 4.**
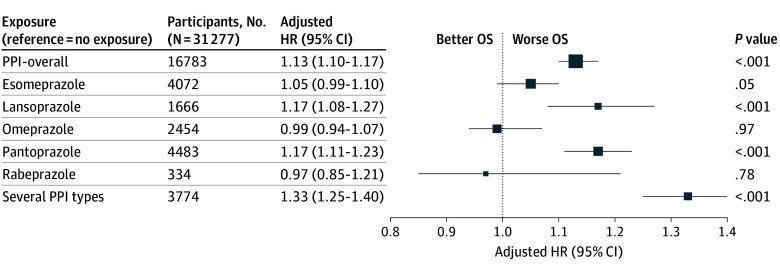
Proton Pump Inhibitor (PPI) Exposure and Overall Survival (OS) HR indicates hazard ratio.

PPI exposure was associated with worse OS (HR, 1.13; 95% CI, 1.10 to 1.17; *P* < .001; absolute difference in probabilities of survival at 24 months, −4.37%; 95% CI, −5.60% to −3.10%) (Figure 1, Figure 4, and eTable 4 in Supplement 1). There was no significant interaction with the immunotherapy regimen (*P* for interaction = .16). Lansoprazole, (HR, 1.17; 95% CI, 1.08-1.27; *P* < .001; absolute difference in probabilities of survival at 24 months, −5.62%; 95% CI, −9.36% to −2.45%), pantoprazole (HR, 1.17; 95 CI, 1.11 to 1.23; *P* < .001; absolute difference in probabilities of survival at 24 months, −4.46%; 95% CI, −6.58% to −2.33%) and several types of PPIs (HR, 1.33; 95% CI, 1.25 to 1.40; *P* < .001; absolute difference in probabilities of survival at 24 months, −10.62%; 95% CI, −12.71% to −8.11%) were associated with worse OS. Results were consistent in sensitivity analyses (eTable 5 in Supplement 1).

## Discussion

In this nationwide cohort study using target trial emulations, exposures to antibiotics, PPIs, and a high dose of steroids were associated with worse OS. The conflicting evidence in the literature can be explained by sampling variation, definition of exposure, and selection and confounding biases. One possible explanation for the antibiotics result is the impact of this treatment on the microbiota. It has been shown that antibiotic exposure within 2 months before the start of immunotherapy alters the gut microbiota composition, leading to an increase in *Enterocloster spp* and other tolerogenic bacteria, which is associated with resistance to immunotherapy.^22^ *Enterocloster spp*, enriched following large spectrum antibiotic exposure, can modulate the mucosal address in cell adhesion molecule 1 (MAdCAM-1) and integrin α4β7 axis, a key gut immune checkpoint involved in cancer immunosurveillance, thereby potentially impairing the efficacy of immunotherapy.^23^ Another explanation could be that these treatments induce more adverse effects. A retrospective study showed that antibiotic exposure might be associated with an increased risk of immune-mediated adverse effects.^24^ It is also possible that antibiotic prescription reflects a more aggressive cancer disease or simply that having an infection itself increases mortality. Moreover, the magnitude of the association was small and could result from unmeasured confounding. We found that negative association of antibiotics with OS was larger in patients who received chemo-immunotherapy, which could be explained by the immunosuppressive effects of chemotherapy worsening the prognosis of infections, for example in case of neutropenia. The antibiotics associated with greater risk, like sulfonamides or penicillins combined with penicillinase inhibitors, are broad-spectrum antibiotics that may have a larger impact on the microbiota, be prescribed for more severe infections, or both. Moreover, sulfonamides are often given as *Pneumocystis* prophylaxis in patients with an immunocompromised condition. Regardless of the underlying reason for the association with worse OS, some antibiotics, such as macrolides and penicillins, were not associated with OS despite being widely used. Macrolides exhibit anti-inflammatory and immunomodulatory effects by modulating cytokine production, reducing neutrophil activity, and influencing gut immune responses, in particular for respiratory disease.^25^ Meta-analysis suggests that penicillin has only minor effects on the intestinal microbiota.^26^ Because avoiding antibiotics in patients with infections or postponing the start of the immunotherapy until a less detrimental window is not an option, these alternatives could be preferred over higher-risk antibiotics when possible. PPIs alter the gut microbiota composition by increasing the presence of oral bacteria, such as Streptococcus species, in the gastrointestinal tract, which has been associated with resistance to immunotherapy.^27^ A retrospective study on immunotherapy in patients with lung cancer, where fecal analyses were conducted, showed that PPI exposure altered the gut microbiota and decreased survival, while fecal transplantation restored immunotherapy efficacy.^28^ Another study revealed that PPI exposure was associated with significantly increased risk of acute kidney injury during immunotherapy.^29^ Outside of patients with cancer, PPIs are often associated with an increase in deleterious adverse events.^30^ However, PPI prescription is also associated with polymedications and comorbidities. Our analysis was adjusted on several medications and comorbidities, so it should be robust. It is unclear why our results differed from one PPI to another. Their indications are similar, as are their pharmacokinetics, except maybe for bioavailability. Steroids are known to inhibit the proliferation and differentiation of T lymphocytes by blocking cluster differention28 pathway stimulation,^31^ so their detrimental effect should be explained by decreasing the immune response. However, steroids are also prescribed to alleviate symptoms in patients with high burden disease and brain metastasis. A higher dose of steroid is often associated with a more aggressive disease and toxicity, which were not available in our models and could have confounded by indication of the association of steroid with OS. In our study, patients with chemotherapy who were exposed to 10 to 20 mg per day had better survival, which could be an artifact of the prescription of antiemetic prophylaxis prescribed with pemetrexed in a population with nonsquamous cancer, good adherence, and good general state.

### Strengths and Limitations

Our study has several strengths. Because of the large sample size, we were able to explore numerous associations with great power and high confidence. Moreover, we employed up-to-date causal analysis methods such as the target trial emulation approach, which strengthens the validity of our results. External validity should be high because we used a nationwide empirical database. However, our results are applicable only to patients who survive the first 2 months of treatment.

There are also several limitations. Reimbursements for exposures of interest were not available during hospitalizations; however, outpatient prescriptions were fully covered. We are confident that this covers the vast majority of exposures because our proportions were at least comparable to, if not higher than, those reported in the literature.^3,4,32^ Information on brain metastases was not available, although we attempted to approximate it using proxies. The indication for the various exposures was not known, leading to uncertain interpretation of the results, particularly for antibiotics and steroids. Despite adjustment, residual confounding may be still present. Additionally, the proposed target trial does not appear ethically feasible in practice, so the only answers that will ever be provided will likely come from observational data.

## Conclusions

In this cohort study of 41 529 patients treated with first line pembrolizumab for an advanced NSLSC, we confirmed the association of OS with exposure to antibiotics, PPIs, and steroids at the beginning of immunotherapy. Antibiotic prescription is mandatory in case of serious infection, but the choice of antibiotic could be oriented toward macrolide or penicillin, drugs without an association with survival. Steroid prescription, if administered at low dose, should not delay or prevent the start of immunotherapy because the dose-dependent negative association with survival was observed at higher dose. PPI prescription, outside of validated indications such as gastric ulcer, should not be proposed to patients with immunotherapy.

## Notes

Supplement 1. **eTable 1.** Specification of the Target Trials #1 (Antibiotics), #2 (Steroids) and #3 (PPI) and Their Observational Emulations
eMethods.
**eTable 2.** Variables Definitions **eFigure 1.** Directed Acrylic Graphs of Antibiotic, Steroid, and PPI Models **eFigure 2.** Plots of Propensity Scores **eFigure 3.** Love Plots of Antibiotic, Steroid, and PPI Models **eFigure 4.** Flow Chart **eTable 3.** Population Description **eTable 4.** Absolute Survival Difference at 24 Months **eFigure 5.** Log Hazards of Steroid Dose **eTable 5.** Sensitivity Analysis

Supplement 2.
Data Sharing Statement
