# Supplementary material for: Concomitant Comedications and Survival With First-Line Pembrolizumab in Advanced Non–Small-Cell Lung Cancer
Source: JAMA Netw Open. 2025 Sep 10;8(9):e2529225. doi: 10.1001/jamanetworkopen.2025.29225 (PMC12423871; doi:10.1001/jamanetworkopen.2025.29225)
Supplement: Supplement 1. — eTable 1. Specification of the Target Trials #1 (Antibiotics), #2 (Steroids) and #3 (PPI) and Their Observational Emulations eMethods. eTable 2. Variables Definitions eFigure 1. Directed Acrylic Graphs of Antibiotic, Steroid, and PPI Models eFigure 2. Plots of Propensity Scores eFigure 3. Love Plots of Antibiotic, Steroid, and PPI Models eFigure 4. Flow Chart eTable 3. Population Description eTable 4. Absolute Survival Difference at 24 Months eFigure 5. Log Hazards of Steroid Dose eTable 5. Sensitivity Analysis [file jamanetwopen-e2529225-s001.pdf]

Supplemental Online Content

Rousseau A, Simon-Tillaux N, Michiels S, et al. Concomitant comedications and survival with first-line pembrolizumab in advanced non–small cell lung cancer. *JAMA Netw. Open*. 2025;8(8):e2529225. doi:0.1001/jamanetworkopen.2025.29225

eTable 1. Specification of the Target Trials #1 (Antibiotics), #2 (Steroids) and #3 (PPI) and Their Observational Emulations
eMethods.
eFigure 1. Directed Acrylic Graphs of Antibiotic, Steroid, and PPI Models
eFigure 2. Plots of Propensity Scores
eFigure 3. Love Plots of Antibiotic, Steroid, and PPI Models
eFigure 4. Flow Chart
eTable 3. Population Description
eTable 4. Absolute Survival Difference at 24 Months
eFigure 5. Log Hazards of Steroid Dose
eTable 5. Sensitivity Analysis

This supplemental material has been provided by the authors to give readers additional information about their work.

© 2025 Rousseau A et al. *JAMA Network Open*.

eTable 1: Specification of the target trials #1 (antibiotics), #2 (steroids) and #3 (PPI) and their observational emulations

Protocol Component	Description of ideal randomized control trials	Description of observational emulations using ATHENA cohort
Eligibility criteria	Inclusion criteria:<br>● Histological or cytological diagnosis of Stage IV NSCLC lacking epidermal growth factor receptor (EGFR)-sensitizing mutation and/or anaplastic lymphoma kinase (ALK) translocation, and received no prior systemic chemotherapy treatment for their metastatic NSCLC<br>● No history of prior malignancy, with the exception of basal cell carcinoma of the skin, superficial bladder cancer, squamous cell carcinoma of the skin, or in situ cervical cancer, or has undergone potentially curative therapy with no evidence of that disease recurrence for 5 years since initiation of that therapy<br>Non-inclusion criteria:<br>● EGFR sensitizing mutation and/or ALK translocation<br>● Has received systemic therapy for the treatment of their	● Any hospitalization or any expenses for a long-term condition identified by ICD-10 lung cancer codes (C33, C34, D02.1, and D02.2)<br>● Being older than 18 years old<br>● Being treated by pembrolizumab in first-line setting.<br>● No onset of a hospitalization with a lung cancer code during the 5 previous years preceding<br>● No same-sex twins or changes in insurance plans<br>● No other cancer diagnosis, identified the previous year prior to the date index<br>● ● For antibiotic (emulated trial #1): no hospitalization for infectious disease 2 months prior to the immunotherapy start<br>● For steroid (emulated trial #2): no hospitalization for autoimmune disease or organ transplantation 12 months prior to the immunotherapy start<br>● For PPI (emulated trial #3): no hospitalization for an

© 2025 Rousseau A et al. *JAMA Network Open*.

	stage IV NSCLC. Completion of treatment with chemotherapy and/or radiation as part of neoadjuvant/adjuvant therapy is allowed as long as therapy was completed at least 6 months prior to the diagnosis of metastatic disease.<br>● Received prior systemic cytotoxic chemotherapy, biological therapy, major surgery within 3 weeks of first dose of study drug; received thoracic radiation therapy of > 30 gray (Gy) within 6 months of first dose of study drug<br>● Received prior therapy with an anti-programmed cell death protein 1 (anti-PD-1), anti-PD-L1, anti-programmed cell death-ligand 2 (anti-PD-L2), anti-CD137 (4-1BB ligand, a member of the Tumor Necrosis Factor Receptor [TNFR] family), or anti-Cytotoxic T-lymphocyte-associated antigen-4 (anti-CTLA-4) antibody (including ipilimumab or any other antibody or drug specifically targeting T-cell co-stimulation or checkpoint pathways)	esophagitis/gastritis/ duodenitis/ulcer and no fibroscopy 6 months prior to the immunotherapy start for PPI’s analysis.

© 2025 Rousseau A et al. *JAMA Network Open*.

	- Active autoimmune disease that has required systemic treatment in past 2 years - Allogenic tissue/solid organ transplant - Interstitial lung disease or pneumonitis that has required oral or IV steroids - Active infection requiring IV systemic therapy	
Treatment strategies	- Experimental: Administration of antibiotic/PPI/steroid treatment at adapted dose in the 42 days (#1 #3), or first month (#2) of pembrolizumab treatment - Comparator : no antibiotic/PPI/steroid treatment in the 42 days (#1 #3), or first month (#2) of pembrolizumab treatment	- Experimental: Antibiotic treatment 60 days before to 42 days after pembrolizumab start (emulated trial #1) - Experimental: Steroid treatment 30 days before to 30 days after pembrolizumab start (emulated trial #2) - Experimental: PPI treatment 60 days before to 42 days after pembrolizumab start (emulated trial #3) - Comparator : no antibiotic/PPI/steroid treatment in the 42 days (#1 #3), or first month (#2) of pembrolizumab treatment
Assignment procedures	Participants are randomized to treatment strategies (experimental or comparator arm) at baseline and are aware of the strategy they are assigned to	- Patients are classified on the basis of the observed treatment initiation during the exposition period. - Randomization is emulated with

© 2025 Rousseau A et al. *JAMA Network Open*.

	(open label).	propensity score analyses and conditionally on baseline covariates selected.<br>● Patients are aware of the strategy they are assigned to.
Follow-up	● Follow-up begins at randomization.<br>● Patients are followed every months for 6 months, then every 3 months for 2 years and the every 6 months for 3 years<br>● Follow-up ends at the earliest of death, loss to follow-up, or administrative end of follow-up.	● Time zero of follow-up is 2 months after the initiation of pembrolizumab<br>● Follow-up ends at the earliest occurrence of death or the latest reimbursed healthcare
Outcome	● Primary: Overall survival (OS) was defined as the time from randomization to death due to any cause. Participants without documented death at the time of the analysis were censored at the date of the last follow-up	● Overall survival (OS) was defined as the time from 2 months after the initiation of pembrolizumab to death from any cause. This outcome is extracted from SNDS database. Participants without documented death at the time of the analysis are censored at the date of the last reimbursed healthcare.
Causal contrast	● Intention-to-treat (ITT) for efficacy	● The association of interest is the hazard ratio. As the database includes information about prescription fill and not adherence, it represents the intention-to-treat

		analysis (as-treated and not on-treatment).
Analysis plan	• Time-to-event distributions are estimated by Kaplan–Meier analysis. Cox proportional-hazards models are used to estimate hazard ratios and test for the significance of time-to-event variables.	• Time-to-event distributions are estimated by Kaplan–Meier analysis.<br>• Hazard ratios, along with their confidence intervals at the 95% level, were estimated from weighted Cox proportional-hazards models with stabilized inverse probability of treatment weights (estimation of the average treatment effect of the whole population)

© 2025 Rousseau A et al. *JAMA Network Open*.

eMethods:

Data source

The French National Health Data System (SNDS) includes demographic and medical data on most outpatient services reimbursed by the National Health Insurance since 2006, encompassing prescribed drugs, patient eligibility for full reimbursement of health care expenses related to specific costly or long-term diseases, and physician visits. It also comprises diagnoses related to hospital admissions and procedures performed during hospitalization. Data collection for reimbursement is nearly comprehensive and automatic. Prescription fills are coded according to the Anatomical Therapeutic Chemical (ATC) classification, diagnoses are registered according to the International Classification of Diseases, 10th Revision (ICD-10) . Variables included demographic data, comorbidities, other medications prescriptions, hospital type and type of treatment (pembrolizumab alone or with chemotherapy). ECOG and PD-L1 tumor status were not available, but patients with altered performance status tends to receive treatment in inpatient clinics and low PDL1 patients receive pembrolizumab plus chemotherapy. Thus, first pembrolizumab in inpatient clinics and chemoimmunotherapy were used as proxies for those variables. Information about brain metastasis was not available, but we used the prescription of radiation therapy and antiepileptic drugs as proxies. Detailed definitions are available in Supplementary Table S2.

Statistical analysis

Propensity score

We first estimate the propensity score for each patient, ie the probability of being exposed to each studied comedications (i.e. antibiotic, steroid or PPI), with a multivariable logistic regression. The introduced covariables in the models were: chemo-immunotherapy (or pemetrexed prescription if the analysis concerned only chemo-immunotherapy population), sex, age at diagnosis, year of diagnosis, type of center, deprivation index, first pembrolizumab in inpatient hospitalization, antibiotics (unless it was the modelized exposure), PPI (unless it was the modelized exposure), steroids (unless it was the modelized exposure), an history of: radiotherapy, antiepileptic, myocardial infarction, heart failure, cerebrovascular disease, peripheral artery disease, chronic respiratory disease, diabetes, kidney failure, renin angiotensin inhibitor, antiplatelet, anticoagulant, diuretic, beta blocker, lipid lowering drug, non-steroidal anti-inflammatory, painkiller, antipsychotic, antidepressant, opiate substitute drug, and thyroid hormone replacement drug. If the exposure could have multiple categories, a multinomial regression model was used to estimate each probability of receiving the different treatment categories (e.g. type of antibiotic exposure), with the no treatment group as reference. These propensity scores were then used to compute stabilized weights by marginal probabilities of being treated for each patient (with additional truncation of the 1% most extreme weights). The first analysis was the association with exposures in the whole population (average treatment effect, ATE). Finally, a Cox proportional hazards model, weighted with these stabilized weights was used to estimate the hazard ratio of exposure.

Steroid dose

Regarding steroids only, the dose-effect relationship on survival was explored by several means: categorization by 10mg/day intervals, trend test and graphical representation of the association of a continuous variable modelized in a Cox model with restricted cubic splines.

© 2025 Rousseau A et al. *JAMA Network Open*.

Sensitivity analyses

We performed several sensitivity analyses: we tested the impact of artificial tears prescription as a negative control of bias in our models. We also modeled all exposures only in patients with at least 1 year of potential follow-up (lookback period).

© 2025 Rousseau A et al. *JAMA Network Open*.

eTable 2: Variables definitions

Variable	Definition
Lung cancer diagnosis	At least one hospitalization or long-term condition with ICD-10 code: C33, C34, D021, D022
First line pembrolizumab	LES code I000392, I000476, I000501<br><br>*If LES code not available, then* :<br><br>First pembrolizumab infusion within 4 months of diagnosis<br><br>*OR* no thoracic surgery and no radio-chemotherapy and 2 or less chemotherapy sessions before first pembrolizumab<br><br>*OR* thoracic surgery and less than 6 chemotherapy sessions within 6 months of surgery before first pembrolizumab<br><br>*OR* radio-chemotherapy and 2 or less chemotherapy sessions after end of radio-chemotherapy before first pembrolizumab
Chemo-immunotherapy	LES code I000476, I000501<br><br>*If LES code not available, then* :<br><br>One infusion of pemetrexed 7 days before to 7 days after first pembrolizumab infusion<br><br>*OR* at least 2 reimbursements of Aprepitant, Netupitant, Rolapitant, Ondansetron, Palonosetron, Dolasetron, Granisetron, Tropisetron 15 days before to 2 months after first pembrolizumab infusion<br><br>*OR* at least 2 reimbursements of Filgrastim, Pegfilgrastim, Lenograstim, Lipegfilgrastim 0 to 2 months after first pembrolizumab infusion
Radiation therapy at baseline	Hospitalization with ICD-10 code Z510 *OR* procedure with CCAM code ZZNL061, ZZNL053, ZZNL064, ZZNL048, ZZNL065, ZZNL062, ZZNL063, ZZNL050, ZZNL054, ZZNL051, ZZNL058, ZZNL052, ZZNL059, ZZNL060, ZZNL049, ZZNL055, ZANL001<br><br>0 to 2 months prior immunotherapy start
Severe myocardial infarction	Hospitalization with ICD-10 code I21;I22;I252;I255<br><br>0 to 12 months prior diagnosis

© 2025 Rousseau A et al. *JAMA Network Open*.

Severe heart failure	Hospitalization with ICD-10 code I110;I130;I132;I50<br><br>0 to 12 months prior diagnosis
Severe cerebrovascular disease	Hospitalization with ICD-10 code G45;G46;H340;I60-I69<br><br>0 to 12 months prior diagnosis
Severe occlusive arteriopathy of the lower limbs	Hospitalization with ICD-10 code I70;I71;I731;I738;I739;I771;I790;I792;K551; K558;K559; Z958;Z959 *OR* or procedure with CCAM code EDAF004, EDAF006, EEAF003, EEAF004, EEAF005, EEAF002, EEAF001, EEAF006<br><br>0 to 12 months prior diagnosis
Chronic respiratory disease	Hospitalization with ICD-10 code I278;I279;J40-J47;J60-J67;J684;J701;J703 *OR* at least 2 reimbursements of ATC R03 drug<br><br>0 to 12 months prior diagnosis
Diabetes	Hospitalization with ICD-10 code E100;E101;E106;E108;E109;E110;E111;E116;E118;E119; E120;E121; E126;E128-E131;E136;E138-E141; E146;E148; E149 *OR* at least 3 reimbursements of ATC A10 drug<br><br>0 to 12 months prior diagnosis
Severe kidney disease	Hospitalization with ICD-10 code I120;I131;N032-N037;N052-N057;N18;N19; N250;Z490;Z491; Z492;Z940;Z992 *OR* procedure with CCAM code Z49, JVJF003, JVJF004, JVJF008, JVJB001, JVJF002, JVJF005, JVJB002<br><br>0 to 12 months prior diagnosis
Severe liver disease	Hospitalization with ICD-10 code I850;I859;I864;I982;K704;K711; K721;K729; K765-K767<br><br>0 to 12 months prior diagnosis
Antibiotic at pembrolizumab start	At least 2 reimbursements of ATC J01 drug 60 days before to 42 days after first pembrolizumab
Steroid at pembrolizumab start	At least 2 reimbursements of ATC H02 drug 1 month before to 1 month after first pembrolizumab
Proton pump inhibitor at pembrolizumab start	At least 2 reimbursements of ATC A02BC drug 60 days before to 42 days after first pembrolizumab

© 2025 Rousseau A et al. *JAMA Network Open*.

Antiepileptic drug at baseline	At least 2 reimbursements of ATC N03 drug<br><br>0 to 2 months prior immunotherapy start
Treatment by renin angiontension system inhibitor (RASI)	At least 2 reimbursements of ATC C09 drug<br><br>0 to 12 months prior diagnosis
Treatment by antiplatelet	At least 2 reimbursements of ATC B01AC drug<br><br>0 to 12 months prior diagnosis
Treatment by anticoagulant	At least 2 reimbursements of ATC B01AA, B01AB, B01AE, B01AF, B01AX drug<br><br>0 to 12 months prior diagnosis
Treatment by diuretic	At least 2 reimbursements of ATC C03 drug<br><br>0 to 12 months prior diagnosis
Treatment by beta blocker	At least 2 reimbursements of ATC C07 drug<br><br>0 to 12 months prior diagnosis
Treatment by non-steroidal anti-inflammatory (NSAI)	At least 2 reimbursements of ATC M01 drug<br><br>0 to 12 months prior diagnosis
Treatment by antipsychotic	At least 2 reimbursements of ATC N05AA, N05AB, N05AC, N05AD, N05AE, N05AF, N05AG, N05AH, N05AL, N05AN, N05AX drug<br><br>0 to 12 months prior diagnosis
Treatment by antidepressant	At least 2 reimbursements of ATC N06AA, N06AB, N06AF, N06AG, N06AX drug<br><br>0 to 12 months prior diagnosis
Treatment by lipid lowering drug	At least 2 reimbursements of ATC C10 drug<br><br>0 to 12 months prior diagnosis
Treatment by thyroid hormone substitute	At least 2 reimbursements of ATC H03AA drug<br><br>0 to 12 months prior diagnosis
Treatment by painkiller	At least 2 reimbursements of ATC C07 drug<br><br>0 to 12 months prior diagnosis
Treatment by opiate substitution therapy	At least 2 reimbursements of ATC N07BC drug<br><br>0 to 12 months prior diagnosis

© 2025 Rousseau A et al. *JAMA Network Open*.

Hospitalization for infectious disease	Hospitalization with ICD-10 code: A00 A01 A02 A03 A04 A05 A06 A07 A08 A09 A15 A16 A17 A18 A19 A20 A21 A22 A23 A24 A25 A26 A27 A28 A30 A31 A32 A33 A34 A35 A36 A37 A38 A39 A40 A41 A42 A43 A44 A45 A46 A47 A48 A49 A50 A51 A52 A53 A54 A55 A56 A57 A58 A59 A60 A61 A62 A63 A64 A65 A66 A67 A68 A69 A70 A71 A72 A73 A74 A75 A76 A77 A78 A79 A80 A81 A82 A83 A84 A85 A86 A87 A88 A89 B50 B51 B52 B53 B54 B55 B56 B57 B58 B59 B60 B61 B62 B63 B64 B65 B66 B67 B68 B69 B70 B71 B72 B73 B74 B75 B76 B77 B78 B79 B80 B81 B82 B83 B85 B86 B87 B88 B89 B95 B96 B97 G00 H60 H62 H65 H66 H67 H68 I33 I38 I39 I400 J00 J01 J02 J03 J04 J05 J06 J12 J13 J14 J15 J16 J17 J18 J85 J86 K65 K67 K750 K830 L00 L01 L02 L03 L04 L05 L06 L07 L08 M00 M01 N300 N390 N41 N70 N71 N72 N73 N74 N75 N76 N77<br><br>0 to 2 months prior immunotherapy start
Esophagitis/gastritis/duodenitis/ ulcer or having a fibroscopy	Hospitalization with ICD-10 code: K219 K20 K210 K221 K253 K259 K263 K269 K290 K291 K292 K293 K294 K295 K296 K297 K298 *OR* or procedure with CCAM code HEQE263 HEQE005 HEQE002 HEQE004<br><br>0 to 6 months prior immunotherapy start
Hospitalization for autoimmune disease or transplantation	Hospitalization with ICD-10 code: D590 D591 D86 D89 E27 G35 G36 G37 H46 I00 I01 I02 J990 K50 K51 L10 L11 L12 L13 L14 M05 M06 M07 M08 M09 M10 M11 M12 M13 M14 M30 M31 M32 M33 M34 M35 M36 M45 M46 M47 M48 M49 M60 M61 M62 M63 M64 M65 M66 M67 M68 M69 M70 M71 M72 M73 M74 M75 M76 M77 M78 M79 N00 N01N04 N05 N10 N11 N12 N13 N14 N15 N16 Z94<br><br>0 to 12 months prior immunotherapy start

Abbreviations: ICD-10= International Classification of Diseases Version 10, CCAM= Classification commune des actes médicaux, ATC= Anatomical Therapeutic Chemical, LES= Liste En Sus

eFigure 1. Directed Acrylic Graphs of Antibiotic, Steroid, and PPI Models

1A: Directed acyclic graph of antibiotic model

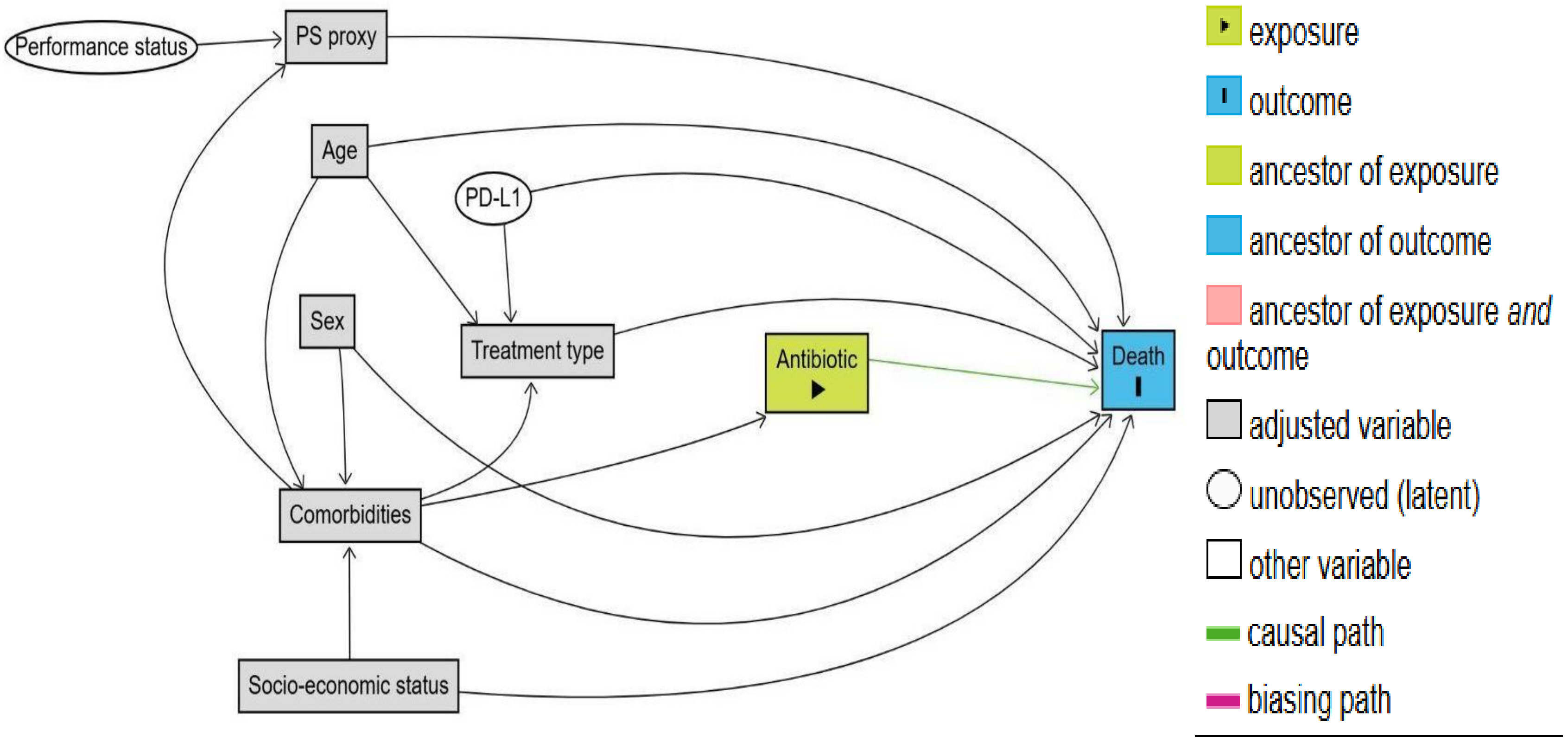

© 2025 Rousseau A et al. *JAMA Network Open*.

1B: Directed acyclic graph of steroid model

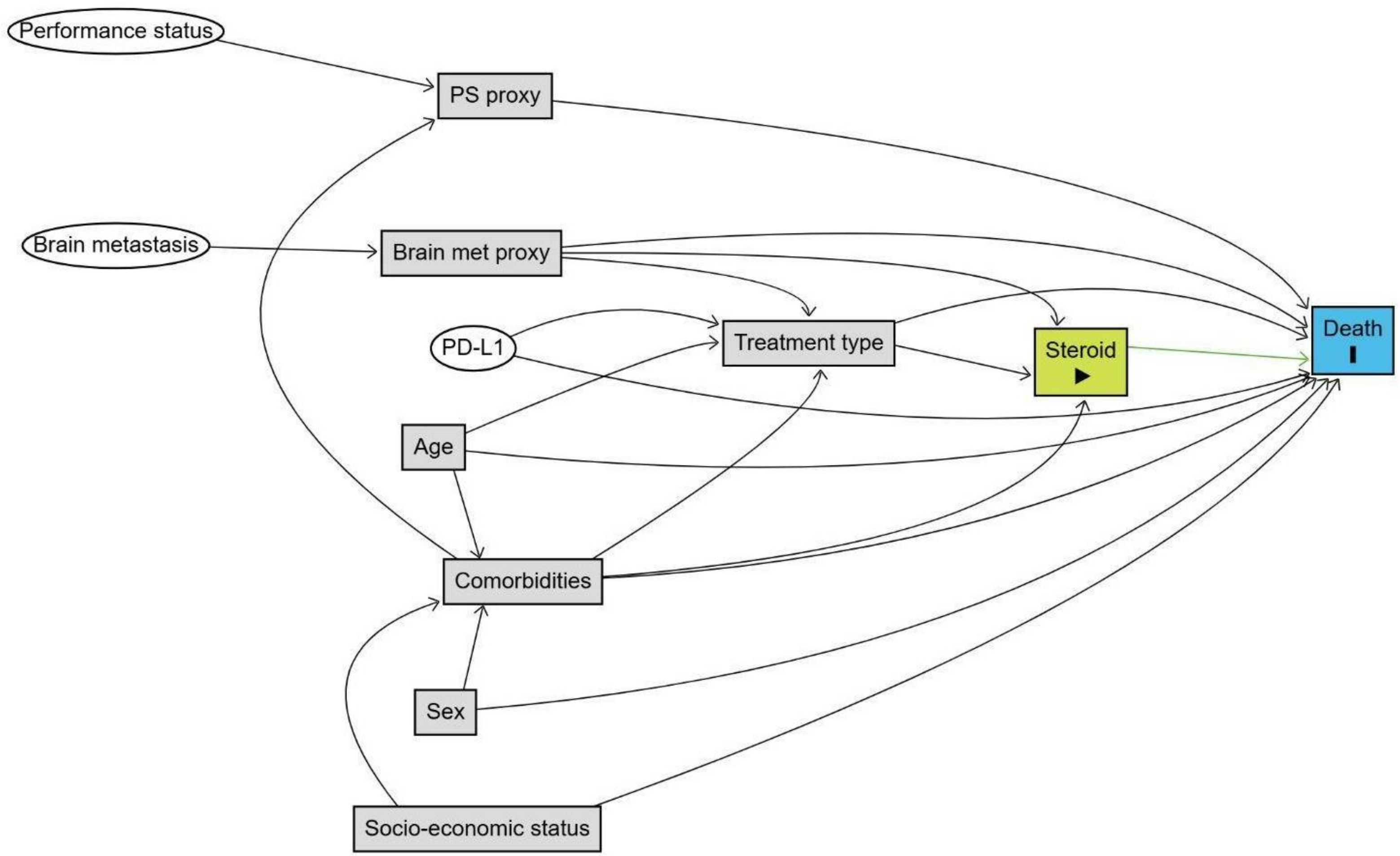

© 2025 Rousseau A et al. *JAMA Network Open*.

1C: Directed acyclic graph of PPI model

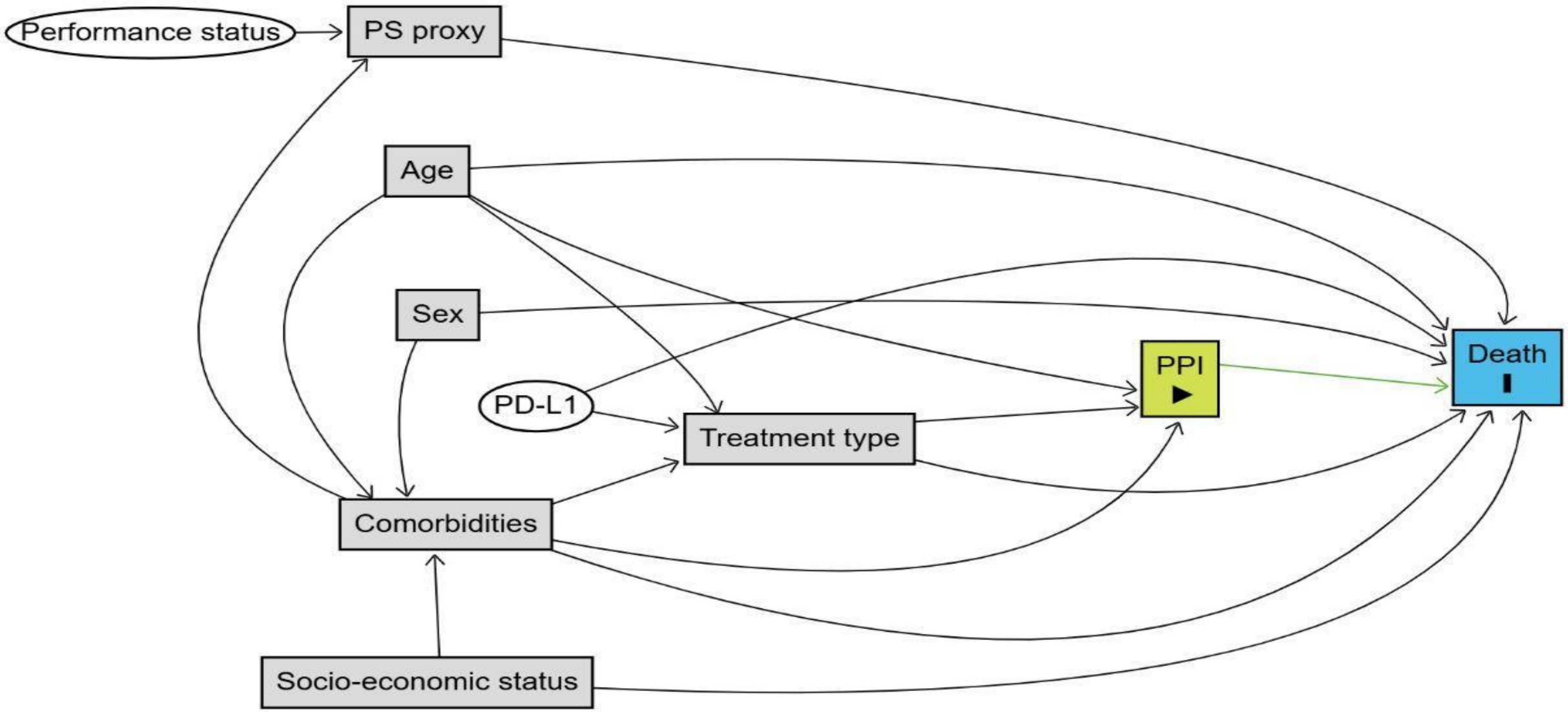

© 2025 Rousseau A et al. *JAMA Network Open*.

eFigure 2. Plots of Propensity Scores

2A: Plot of propensity score of antibiotic

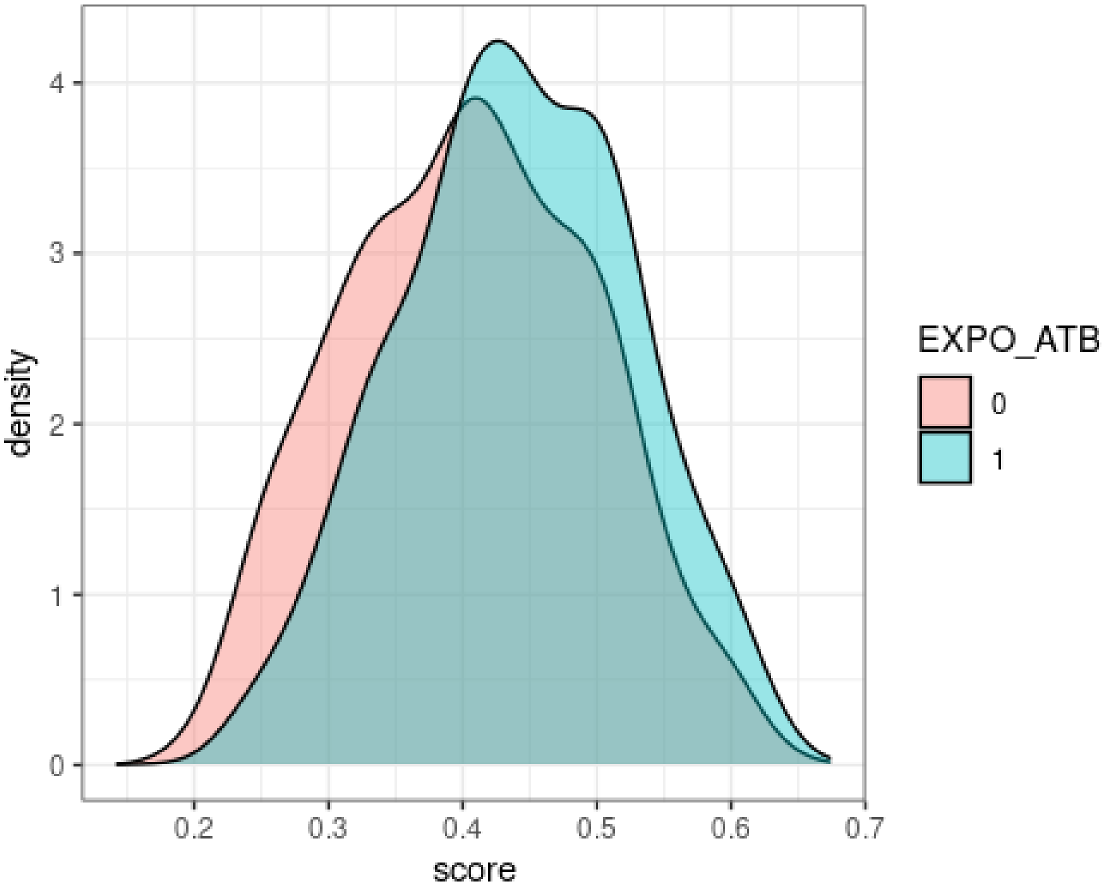

© 2025 Rousseau A et al. *JAMA Network Open*.

<u>2B:</u> Plot of propensity score of steroid

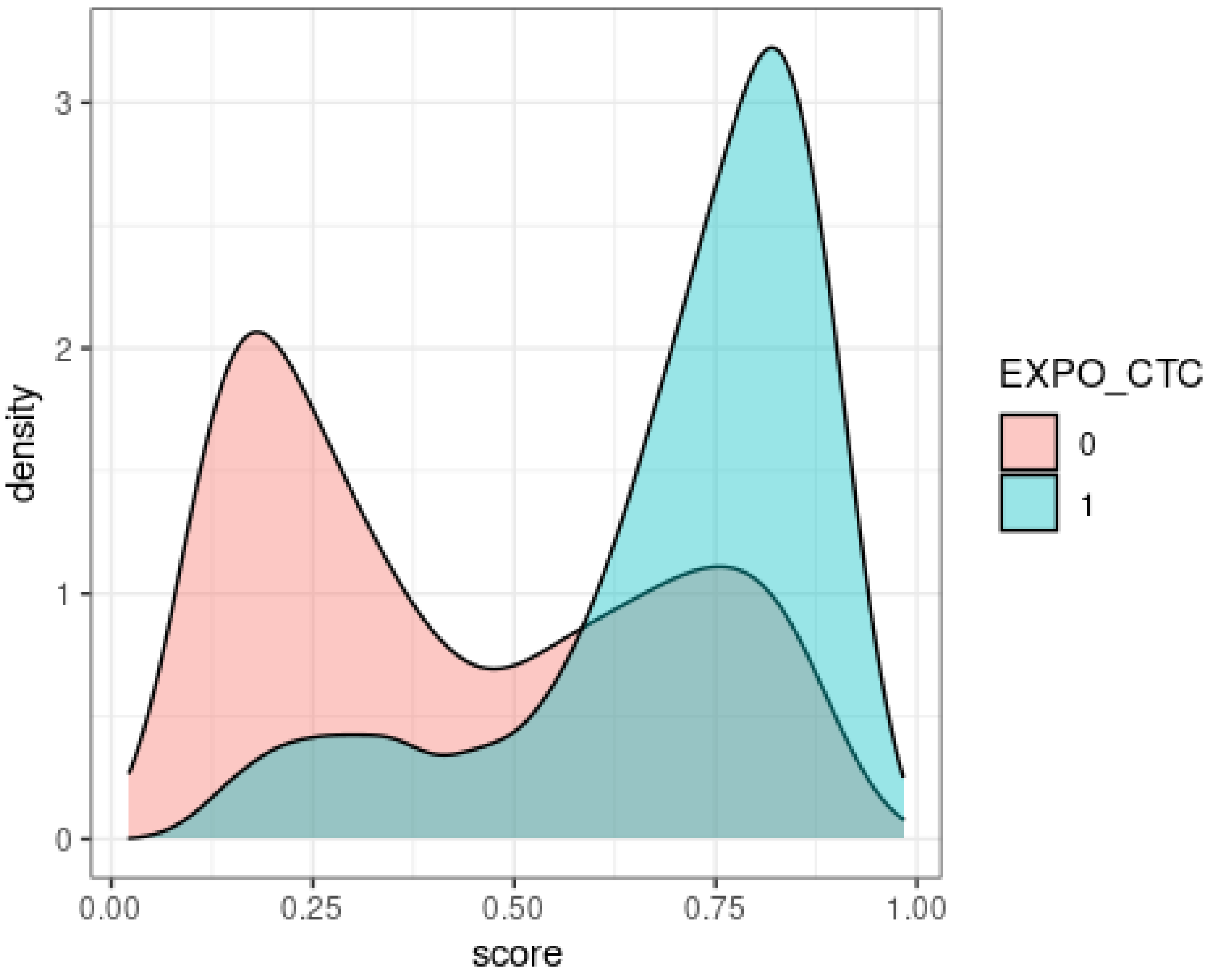

© 2025 Rousseau A et al. *JAMA Network Open*.

2C: Plot of propensity score of PPI

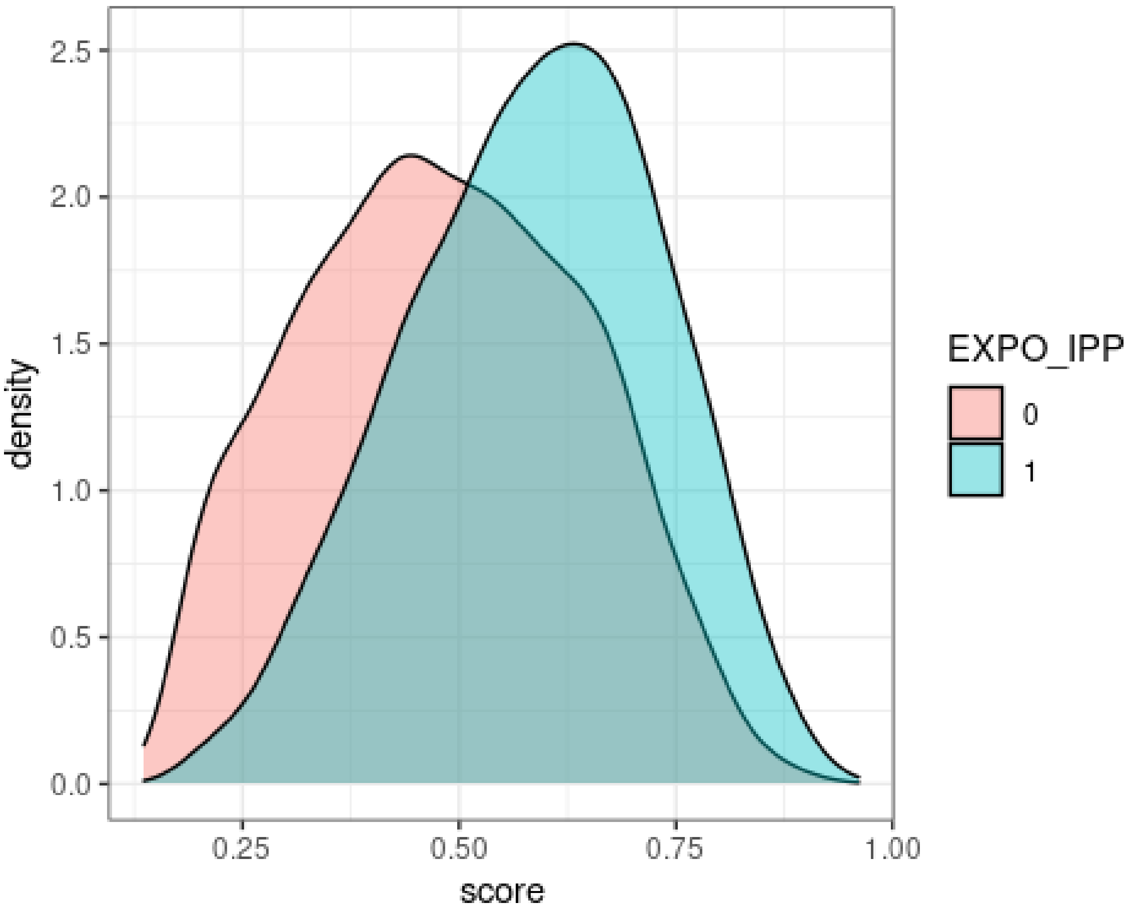

© 2025 Rousseau A et al. *JAMA Network Open*.

eFigure 3. Love Plots of Antibiotic, Steroid, and PPI Models

3A: Love plot of antibiotic model, naive population in *pink* and weighted population in *purple*

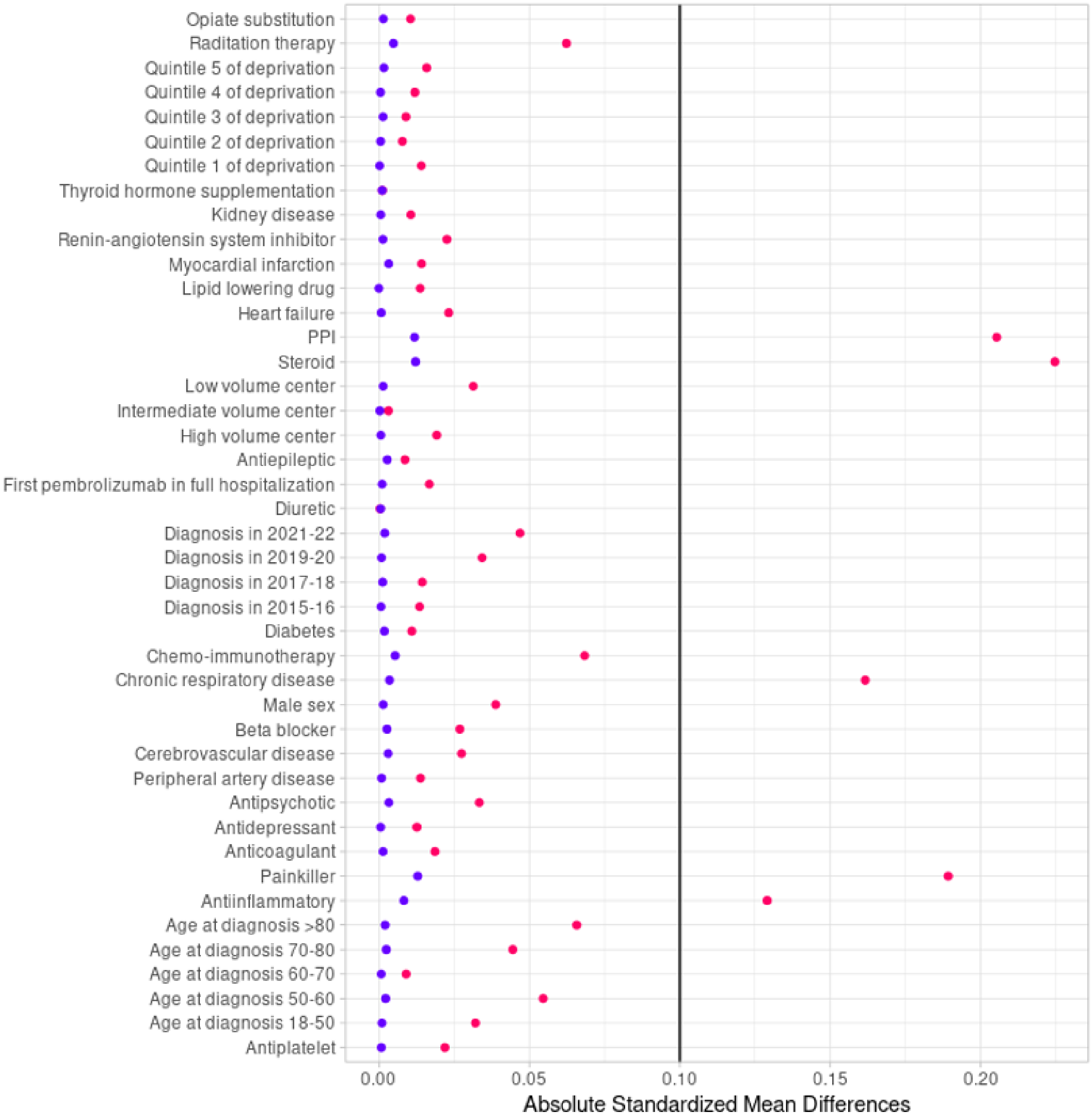

© 2025 Rousseau A et al. *JAMA Network Open*.

<u>3B:</u> Love plot of steroid model, naive population in *pink* and weighted population in *purple*

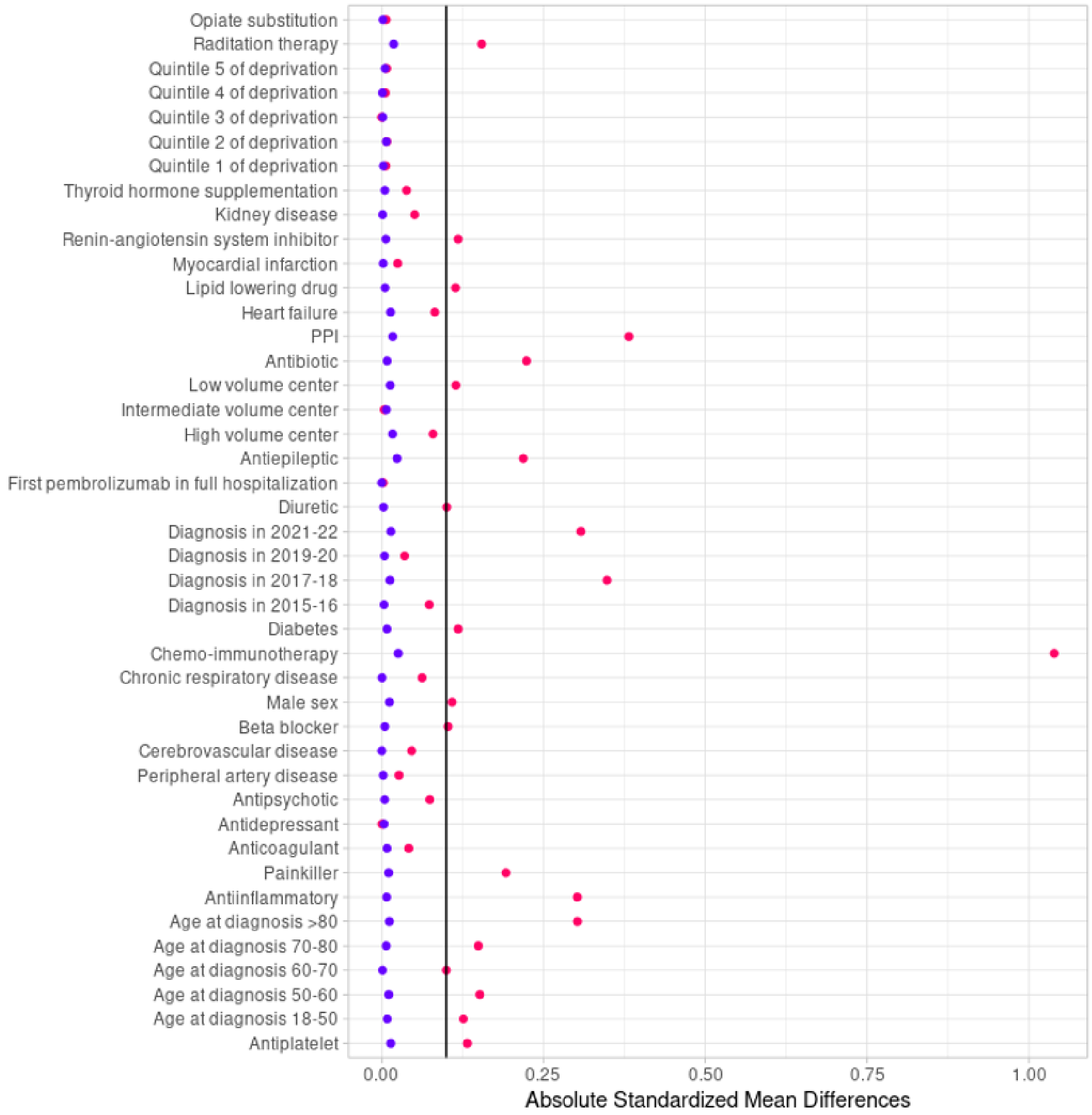

© 2025 Rousseau A et al. *JAMA Network Open*.

<u>3C:</u> Love plot of PPI model, naive population in *pink* and weighted population in *purple*

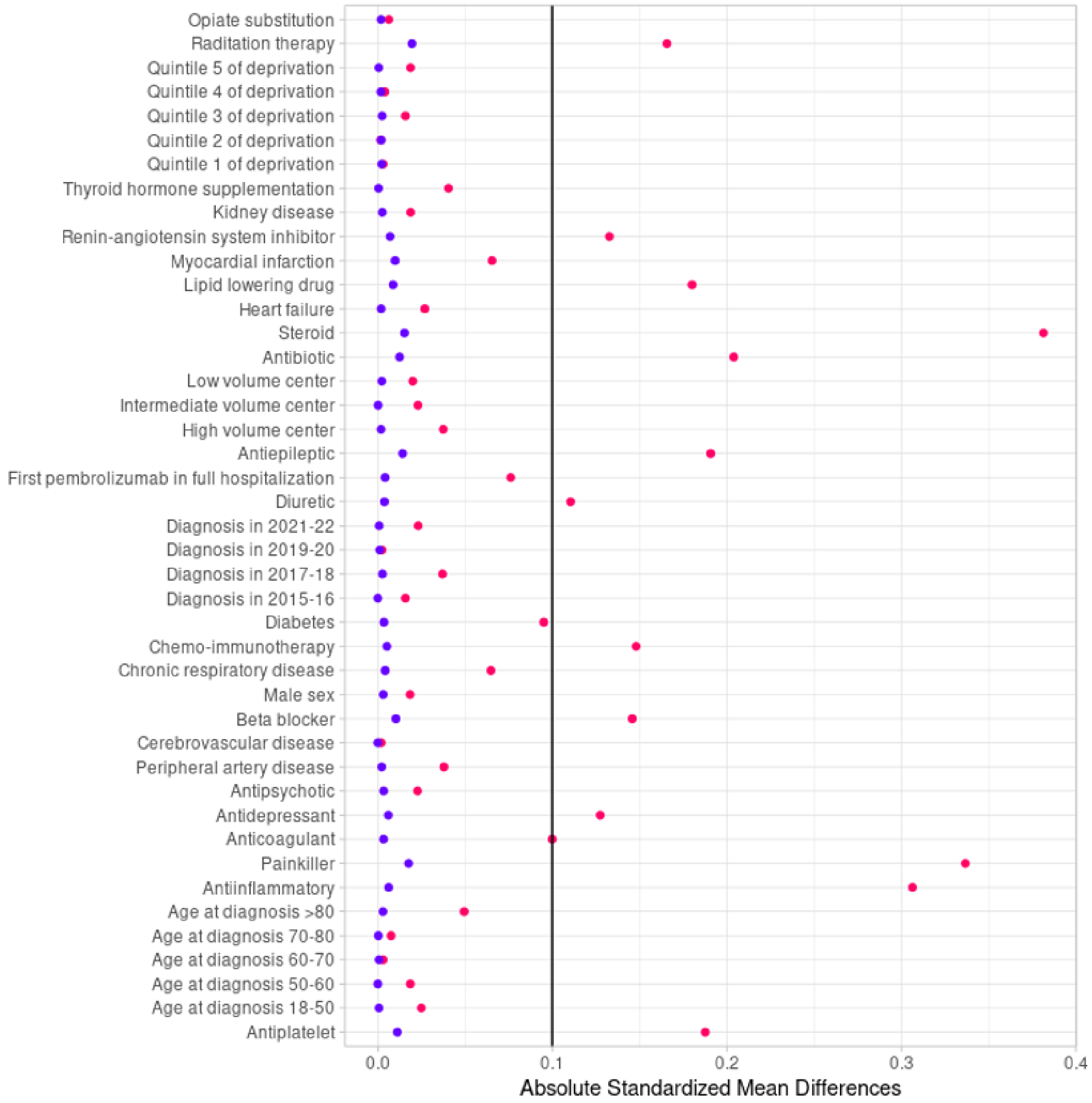

© 2025 Rousseau A et al. *JAMA Network Open*.

eFigure 4: Flow chart

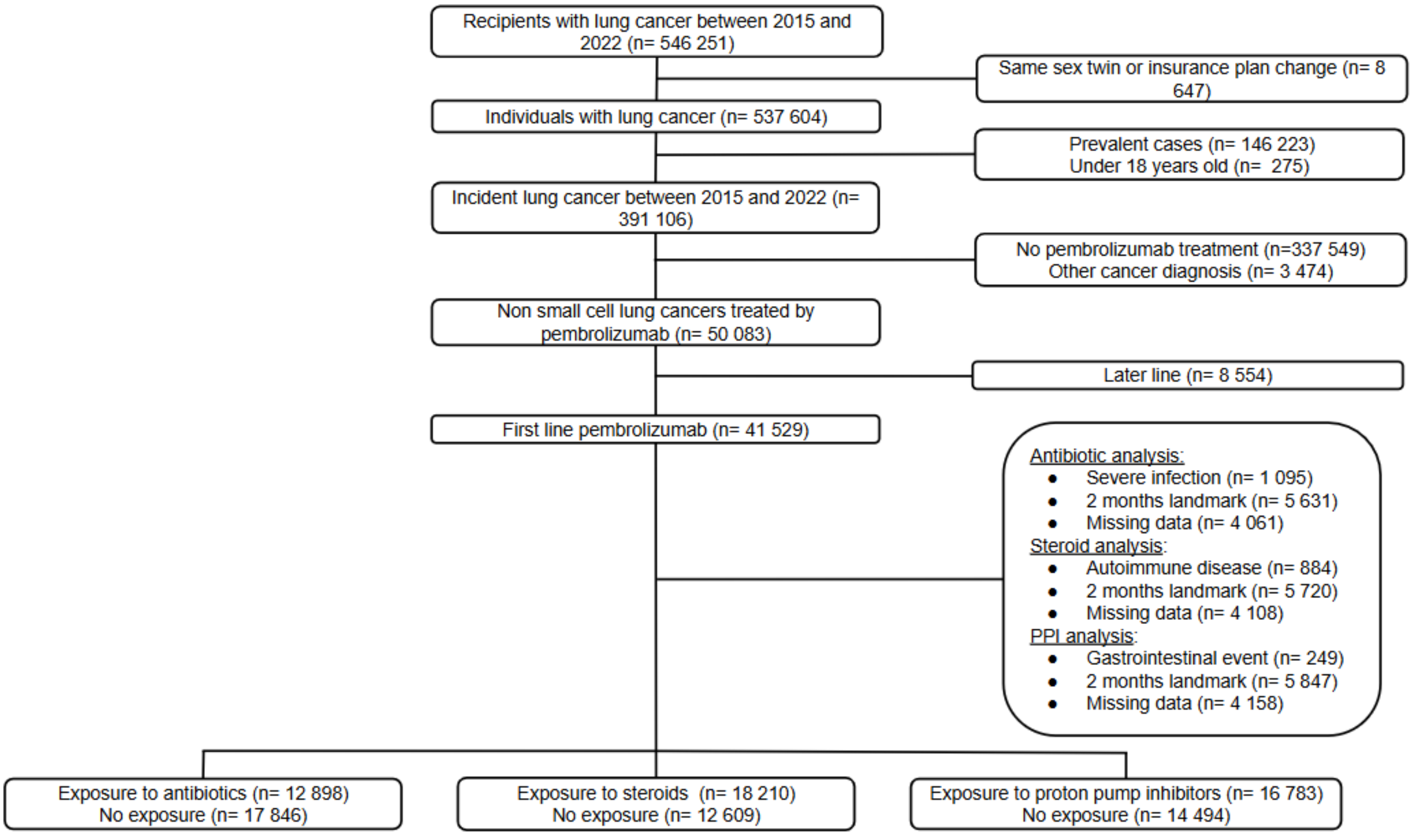

© 2025 Rousseau A et al. *JAMA Network Open*.

eTable 3: Population description

	Overall (N=41531)
**Sex**	
Female	13705 (33.0%)
Male	27826 (67.0%)
**Age at diagnosis**	
Mean (SD)	64.6 (9.87)
Median [Min, Max]	65.0 [19.0, 97.0]
**Age category**	
[18,50)	2849 (6.9%)
[50,60)	9242 (22.3%)
[60,70)	16458 (39.6%)
[70,80)	10345 (24.9%)
[80,97]	2637 (6.3%)
**Year of diagnosis**	
2015-16	920 (2.2%)
2017-18	5799 (14.0%)
2019-20	14586 (35.1%)
2021-22	20226 (48.7%)
**Type of hospital**	
High volume	17467 (42.1%)
Intermediate volume	17792 (42.8%)

© 2025 Rousseau A et al. *JAMA Network Open*.

	Overall (N=41531)
Low volume	6272 (15.1%)
**Deprivation index**	
First quintile	6055 (14.6%)
Second quintile	7450 (17.9%)
Third quintile	7531 (18.1%)
Fourth quintile	7946 (19.1%)
Fifth quintile	7616 (18.3%)
Missing	4933 (11.9%)
**Strategy**	
Pembrolizumab alone	14835 (35.7%)
Pembrolizumab plus chemotherapy	26696 (64.3%)
**Pemetrexed treatment**	
Without pemetrexed	21510 (51.8%)
With pemetrexed	20021 (48.2%)
**Type of first hospitalization**	
Day hospital	29803 (71.8%)
Inpatient hospitalization	11723 (28.2%)
Missing	5 (0.0%)
**Radiation therapy at baseline**	
No	38312 (92.2%)
Yes	3219 (7.8%)

© 2025 Rousseau A et al. *JAMA Network Open*.

	Overall (N=41531)
**Antiepileptic at baseline**	
No	38879 (93.6%)
Yes	2652 (6.4%)
**Exposition to antibiotics at baseline**	
No	23967 (57.7%)
Yes	17564 (42.3%)
**Exposition to steroid at baseline**	
No	18286 (44.0%)
Yes	23245 (56.0%)
**Exposition to PPI at baseline**	
No	19689 (47.4%)
Yes	21842 (52.6%)
**Severe myocardial infarction at diagnosis**	
No	41214 (99.2%)
Yes	317 (0.8%)
**Severe heart failure at diagnosis**	
No	41235 (99.3%)
Yes	296 (0.7%)
**Severe cerebrovascular disease at diagnosis**	
No	41029 (98.8%)
Yes	502 (1.2%)

© 2025 Rousseau A et al. *JAMA Network Open*.

	Overall (N=41531)
**Severe peripheral artery disease at diagnosis**	
No	41021 (98.8%)
Yes	510 (1.2%)
**Chronic respiratory disease at diagnosis**	
No	30220 (72.8%)
Yes	11311 (27.2%)
**Diabetes at diagnosis**	
No	36335 (87.5%)
Yes	5196 (12.5%)
**Severe liver disease at diagnosis**	
No	41515 (100.0%)
Yes	16 (0.0%)
**Severe kidney disease at diagnosis**	
No	41436 (99.8%)
Yes	95 (0.2%)
**Renin-angiotensin system inhibitor at diagnosis**	
No	27282 (65.7%)
Yes	14249 (34.3%)
**Antiplatelet at diagnosis**	
No	29347 (70.7%)
Yes	12184 (29.3%)

© 2025 Rousseau A et al. *JAMA Network Open*.

	Overall (N=41531)
**Lipid lowering drug at diagnosis**	
No	26966 (64.9%)
Yes	14565 (35.1%)
**Anticoagulant at diagnosis**	
No	35748 (86.1%)
Yes	5783 (13.9%)
**Diurectic at diagnosis**	
No	37032 (89.2%)
Yes	4499 (10.8%)
**Beta blocker at diagnosis**	
No	32765 (78.9%)
Yes	8766 (21.1%)
**Non-steroidal anti-inflammatory at diagnosis**	
No	27959 (67.3%)
Yes	13572 (32.7%)
**Antipsychotic at diagnosis**	
No	40253 (96.9%)
Yes	1278 (3.1%)
**Antidepressant at diagnosis**	
No	35154 (84.6%)
Yes	6377 (15.4%)

© 2025 Rousseau A et al. *JAMA Network Open*.

	Overall (N=41531)
**Thyroid hormone replacement drug at diagnosis**	
No	38700 (93.2%)
Yes	2831 (6.8%)
**Painkiller at diagnosis**	
No	10371 (25.0%)
Yes	31160 (75.0%)
**Opiate substitute drug at diagnosis**	
No	41203 (99.2%)
Yes	328 (0.8%)

© 2025 Rousseau A et al. *JAMA Network Open*.

eTable 4: Absolute survival difference at 24 months

	**Absolute survival difference at 24 months (Δ24), %, [95%CI]**
Antibiotic	-2.53 [-3.82 – -1.22]
Antibiotic - pembrolizumab	-0.77 [-2.89 – 1.23]
Antibiotic - chemo-immunotherapy	-3.65 [-5.43 – -2.04]
Fluoroquinolone - pembrolizumab	-0.62 [-8.17 - 6.34]
Macrolide - pembrolizumab	-1.27 [-6.18 - 4.56]
Other antibiotic - pembrolizumab	1.47 [-10.6 -13.5]

© 2025 Rousseau A et al. *JAMA Network Open*.

Other beta lactam - pembrolizumab	-8.47 [-18.0 - -1.49]
Penicillin - pembrolizumab	0.86 [-4.32 - 5.38]
Penicillin + penicillinase inhibitor - pembrolizumab	-1.17 [-4.67 - 2.21]
Several antibiotic types - pembrolizumab	1.16 [-2.29 - 4.61]
Sulfonamid - pembrolizumab	-19.3 [-31.1 - -7.09]
Fluoroquinolone - chemo-immunotherapy	-8.55 [-15.2 - -2.94]
Macrolide - chemo-immunotherapy	1.62 [-2.91 - 5.98]
Other antibiotic - chemo-immunotherapy	3.73 [-5.16 - 11.2]

© 2025 Rousseau A et al. *JAMA Network Open*.

Other beta lactam - chemo-immunotherapy	-6.21 [-16.0 - 0.75]
Penicillin - chemo-immunotherapy	0.08 [-4.37 - 3.56]
Penicillin + penicillinase inhibitor - chemo-immunotherapy	-4.69 [-7.71 - -1.84]
Several antibiotic types - chemo-immunotherapy	-4.61 [-7.18 - -2.34]
Sulfonamid - chemo-immunotherapy	-12.7 [NA - -5.44]
Steroid	-0.001 [-1.54 - 1.72]
Steroid - pembrolizumab	-6.00 [-8.42 - -3.44]
Steroid - chemo-immunotherapy	3.53 [1.39 - 5.77]

© 2025 Rousseau A et al. *JAMA Network Open*.

Glucosteroid - pembrolizumab	-4.44 [-7.36 - -2.03]
Hydrocortisone - pembrolizumab	0.90 [NA - 11.3]
Several steroid types - pembrolizumab	-15.3 [-18.0 - -2.06]
1-10 mg/d - pembrolizumab	-6.13 [-18.8 - 2.43]
10-20 mg/d - pembrolizumab	-1.63 [-8.83 - 5.69]
20-30 mg/d - pembrolizumab	-5.30 [-11.8 - 1.30]
30-40 mg/d - pembrolizumab	-15.9 [-23.9 - -3.98]
40-50 mg/d - pembrolizumab	-16.8 [-27.0 - -6.31]

© 2025 Rousseau A et al. *JAMA Network Open*.

50-60 mg/d - pembrolizumab	-17.4 [-49.3- -1.50]
60-70 mg/d - pembrolizumab	-23.2 [-46.9 - -9.68]
70-80 mg/d - pembrolizumab	-18.4 [NA - 6.22]
over 80 mg/d - pembrolizumab	-27.9 [-42.5 - -13.7]
Glucosteroid - chemo-immunotherapy	2.39 [1.38 - 6.63]
Hydrocortisone - chemo-immunotherapy	NA
Several steroid types - chemo-immunotherapy	-3.20 [-7.66 - 3.34]

© 2025 Rousseau A et al. *JAMA Network Open*.

1-10 mg/d - chemo-immunotherapy	-1.54 [-8.18 - 5.53]
10-20 mg/d - chemo-immunotherapy	1.54 [-0.93 - 3.74]
20-30 mg/d - chemo-immunotherapy	-2.78 [-5.67 - 0.13]
30-40 mg/d - chemo-immunotherapy	-6.47 [-12.7 - -0.64]
40-50 mg/d - chemo-immunotherapy	-9.56 [-15.2 - -4.19]
50-60 mg/d - chemo-immunotherapy	-4.34 [-17.1 - 4.30]
60-70 mg/d - chemo-immunotherapy	-14.1 [-24.7 - -7.37]

© 2025 Rousseau A et al. *JAMA Network Open*.

70-80 mg/d - chemo-immunotherapy	-28.8 [NA - -11.6]
over 80 mg/d - chemo-immunotherapy	-18.5 [-27.0 - -11.6]
PPI	-4.37 [-5.60 - -3.10]
Esomeprazole	-3.21 [-5.13 - -0.90]
Lansoprazole	-5.62 [-9.36 - -2.45]
Omeprazole	0.95 [-2.06 - 3.34]
Pantoprazole	-4.46 [-6.58 - -2.33]
Rabeprazole	0.45 [-8.56 - 5.96]
Several PPI	-10.6 [-12.7 - -8.11]

© 2025 Rousseau A et al. *JAMA Network Open*.

eFigure 5. Log Hazards of Steroid Dose

5A: Log hazard of steroid dose in pembrolizumab alone population

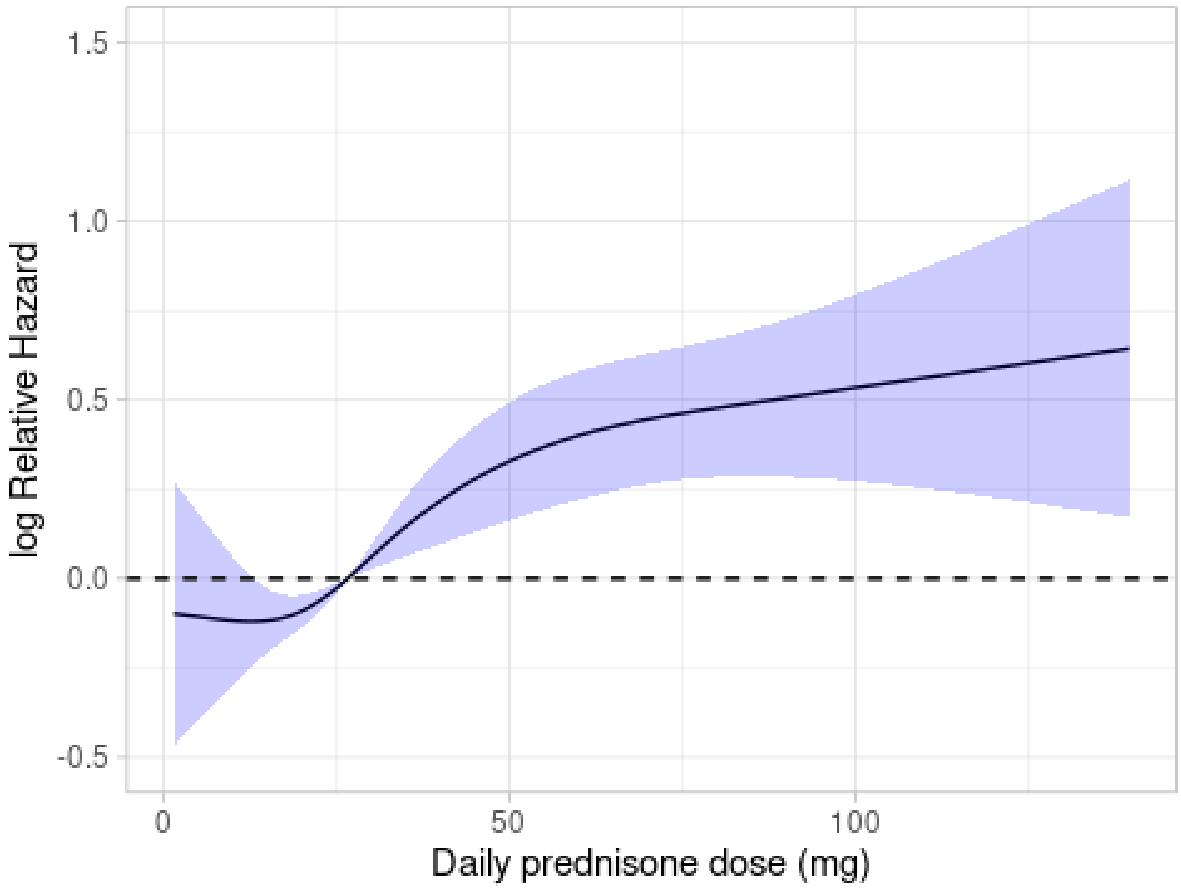

© 2025 Rousseau A et al. *JAMA Network Open*.

5B: Log hazard of steroid dose in pembrolizumab plus chemotherapy population

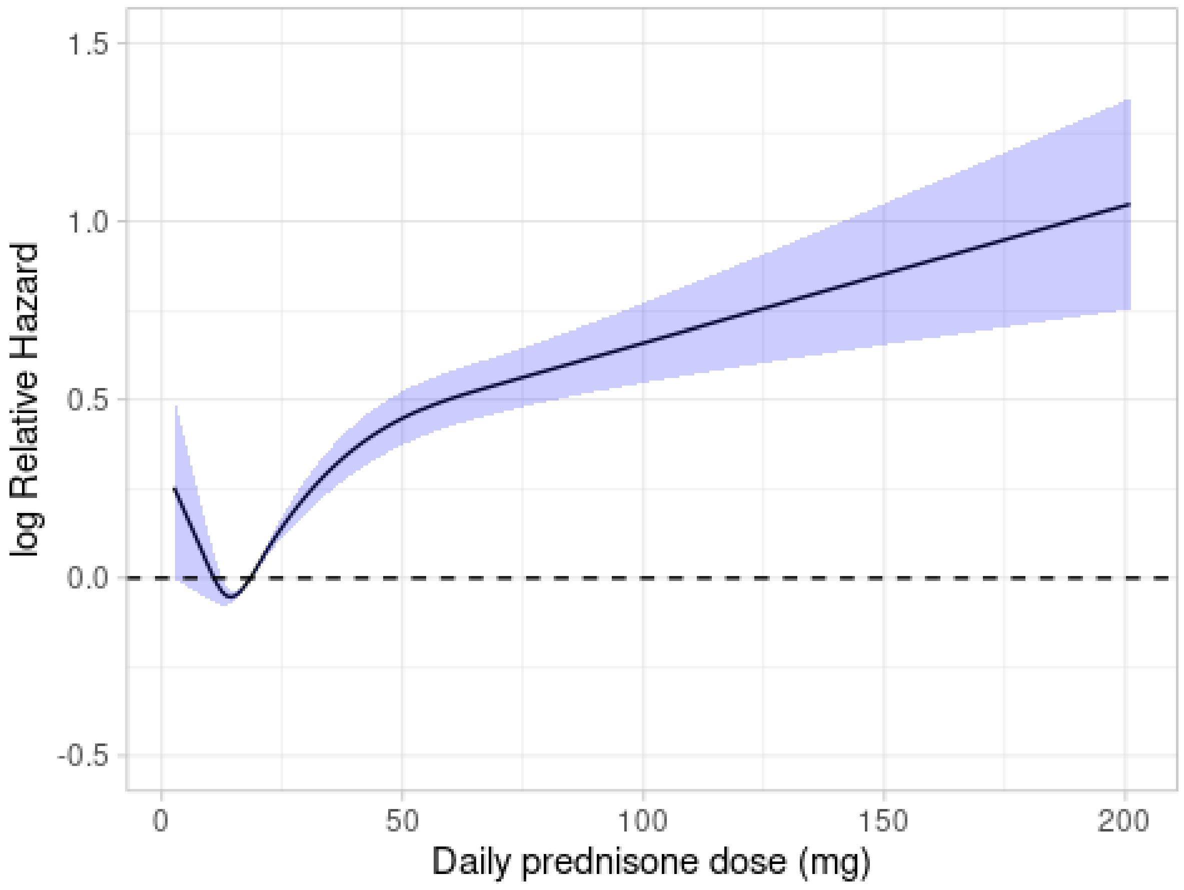

© 2025 Rousseau A et al. *JAMA Network Open*.

eTable 5: sensitivity analyses

Exposure	HR with artificial tear (negative control) in model	HR of artificial tear (negative control) in model	HR in population with at least 1 year of follow up	HR in main analysis
Antibiotic	1.08 [1.05-1.12]	1.02 [0.95-1.11]	1.08 [1.04-1.12]	1.08 [1.05-1.12]
Steroid	0.98 [0.95-1.02]	1.07 [0.96-1.18]	1.00 [0.96-1.04]	0.98 [0.95-1.02]
PPI	1.13 [1.10-1.17]	1.02 [0.94-1.11]	1.14 [1.10-1.18]	1.13 [1.10-1.17]

© 2025 Rousseau A et al. *JAMA Network Open*.
