# Supplementary material for: Concomitant Comedications and Survival With First-Line Pembrolizumab in Advanced Non–Small-Cell Lung Cancer
Source: JAMA Netw Open. 2025 Sep 10;8(9):e2529225. doi: 10.1001/jamanetworkopen.2025.29225 (PMC12423871; doi:10.1001/jamanetworkopen.2025.29225)
Supplement: Supplement 2. — Data Sharing Statement [file jamanetwopen-e2529225-s002.pdf]

Data Sharing Statement

Rousseau. Concomitant Comedications and Survival With First-Line Pembrolizumab in Advanced Non–Small-Cell Lung Cancer. *JAMA Netw Open*. Published August 27, 2025. doi:10.1001/jamanetworkopen.2025.29225

Data
Data available: No

Additional Information
Explanation for why data not available: The database is owned by french government and cannot be shared openly
